# Small molecule anionophores promote transmembrane anion permeation matching CFTR activity

**DOI:** 10.1038/s41598-018-20708-3

**Published:** 2018-02-08

**Authors:** Elsa Hernando, Valeria Capurro, Claudia Cossu, Michele Fiore, María García-Valverde, Vanessa Soto-Cerrato, Ricardo Pérez-Tomás, Oscar Moran, Olga Zegarra-Moran, Roberto Quesada

**Affiliations:** 1 Departamento de Química, Universidad de Burgos, 09001 Burgos, Spain; 2 U.O.C. Genetica Medica, Instituto Giannina Gaslini, Genoa, Italy; 3 Istituto di Biofisica, CNR, Genoa, Italy; 4 Cancer Cell Biology Research Group, Department of Pathology and Experimental Therapeutics, Faculty of Medicine, University of Barcelona, Barcelona, Spain

## Abstract

Anion selective ionophores, anionophores, are small molecules capable of facilitating the transmembrane transport of anions. Inspired in the structure of natural product prodigiosin, four novel anionophores 1a-d, including a 1,2,3-triazole group, were prepared. These compounds proved highly efficient anion exchangers in model phospholipid liposomes. The changes in the hydrogen bond cleft modified the anion transport selectivity exhibited by these compounds compared to prodigiosin and suppressed the characteristic high toxicity of the natural product. Their activity as anionophores in living cells was studied and chloride efflux and iodine influx from living cells mediated by these derivatives was demonstrated. These compounds were shown to permeabilize cellular membranes to halides with efficiencies close to the natural anion channel CFTR at doses that do not compromise cellular viability. Remarkably, optimal transport efficiency was measured in the presence of pH gradients mimicking those found in the airway epithelia of Cystic Fibrosis patients. These results support the viability of developing small molecule anionophores as anion channel protein surrogates with potential applications in the treatment of conditions such as Cystic Fibrosis derived from the malfunction of natural anion transport mechanisms.

## Introduction

Ion transport across biological membranes is a tightly regulated event involved in many cellular processes. The study of these phenomena has been central to supramolecular chemistry and many classical examples of supramolecular complexes and processes involved for instance macrocyclic polyethers capable of facilitating transmembrane cation transport^1,2^. More recently, anion selective ionophores, anionophores, have been studied and a number of synthetic anionophores have been reported in the literature along with insights into the design, function and applications of these compounds^3–6^.

**Figure 1:**
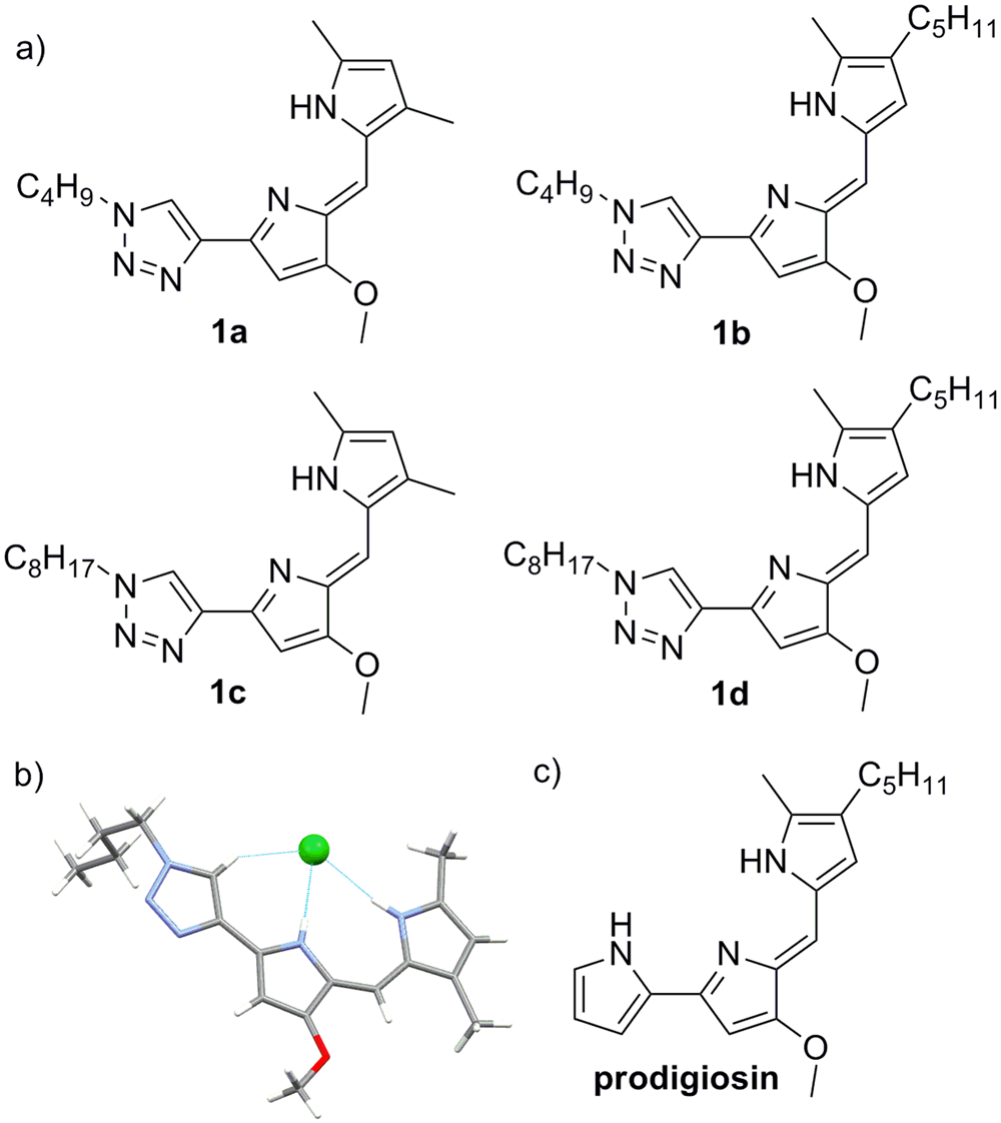
Molecular structure of the anionophores. (**a**) Chemical structure of the compounds **1a-d**. (**b**) Representation of the structure of compound **1a**.HCl obtained from X-ray diffraction. (**c**) Structure of the natural product prodigiosin.

In particular we are interested in the potential biological applications of anionophores^7^. In principle, an active anionophore could impact the cellular homeostasis facilitating anion transport. Changes in the intracellular concentrations of anions affect concentrations gradients, membrane potentials and pH levels, and these changes have been related to cytotoxicity and induced apoptosis^8–10^. In fact, one of the leading examples of anionophores are the prodiginines, a family of bacterial secondary metabolites displaying intriguing pharmacological properties^11,12^. Prodigiosin itself (Fig. 1c) is a highly toxic tripyrrole red pigment. Its biological role can be described as a bacterial weapon used by producing microorganisms (such as *Serratia marcescens*) to eliminate potential competitor microorganisms from a favorable ecological niche. The cytotoxic activity of these compounds is related to their ionophoric activity^13,14^, and a related derivative, obatoclax, has shown potential as anticancer agent in the clinic^15^. Indeed, we and others have studied the cytotoxicity of anionophores and their potential applications as antitumor chemotherapeutics delineating the relationships between their ionophoric activity and the triggered cellular mechanism associated to their action^16–21^.

An equally important potential application for this class of compounds would be as minimalist mimics of transmembrane protein channels^22^. Since the control of ion fluxes at the cellular level is of paramount importance, impairment of the function of the proteins involved in this process often results in serious conditions^23^. A number of diseases are related to the malfunction or lack of membrane proteins facilitating the transport of anions (for example Bartter’s syndrome, Dent’s disease or most notably cystic fibrosis). It is proposed that replacing the missing activity of these proteins could be a viable therapeutic approach for these conditions. Cystic fibrosis (CF) is a genetic disease caused by a mutation of the gene coding for the protein CFTR (cystic fibrosis transmembrane conductance regulator). CFTR is a transmembrane channel that selectively transports chloride and bicarbonate in the apical membrane of epithelium. More than 2000 mutations, classified in six classes according to the effect of the mutation, can result in the lack of CFTR protein synthesis, trafficking and folding defects, or no-functional proteins. Nowadays, two drugs developed by the pharmaceutical company Vertex, ivacaftor and lumacaftor, have been approved for treatment of CF^24,25^. However, the therapeutic results dramatically depend on the mutation harbored by the patient and little therapeutic value has been proved for the most prevalent mutations among CF patients. Using anionophores to replace the defective CFTR activity has in principle the advantage of being a therapeutic strategy independent of the specific mutation carried by the patient. In order to show therapeutic value, the drug candidates should be capable of facilitating significant anion transport at doses that do not compromise the cell viability^26^. Exploring this concept we present here a new family of synthetic compounds inspired in the naturally occurring prodiginines which are highly efficient transmembrane carriers functioning in living cells and displaying moderate cytotoxicity.

## Results

### Synthesis and interaction with anions

Compounds **1a**-**d** (Fig. 1) were obtained in good yields as stable, red-orange solids in the form of hydrochloric acid salts. The polarized C-H group of the triazole heterocycle is a good hydrogen bond donor group that has been included in a number anion receptor designs^27–29^. We expected to obtain molecules with a similar hydrogen bonding cleft than prodiginines yet with different character and displaying an additional modification point such as the triazole substituent to modulate properties such as overall lipophilicity of the molecule. We coined these derivatives as “click prodiginines”.

The ability of these compounds to interact with anions was evident from the ^1^H NMR data (Supplementary Fig. 1). Characteristic deshielding of both N-H and triazole C-H signals were observed. Further evidence came from the solid structure of the hydrochloric salt of **1a** (Fig. 1b). The tris-heterocyclic structure is essentially flat and chloride anion was found interacting with the hydrogen cleft defined by the dypirromethene and the triazole moieties. Both N-H^….^Cl bond distances (3.133 Å, 3.096 Å) and angles (177.19°, 172.14°) as well as C-H^….^Cl bond distance (3.472 Å) and angle (152.13°) are consistent with a strong hydrogen bond interaction. ^1^H NMR spectra showed the existence of different conformational equilibriums in solution depending of the nature of the counter anion and solvent used (Supplementary characterization data).

**Figure 2:**
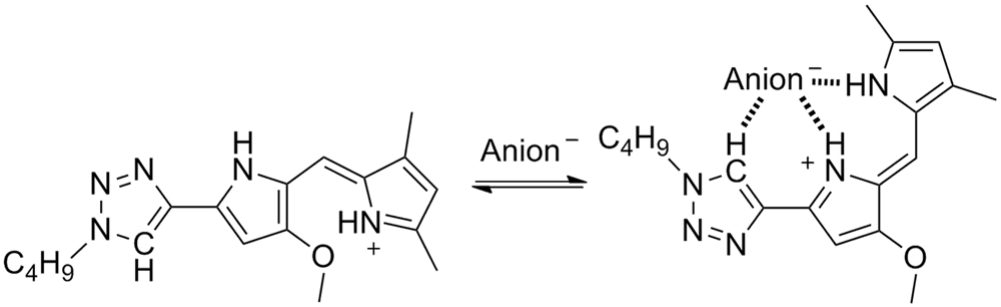
Computed structures for the complexation of compound **1a**.H^+^ and anions.

To explore the energetics of binding of different anions and compound **1a**, we carried out density functional theory (DFT) calculations in different media (gas phase, chloroform, DMSO and water) in order to obtain data across a range of relative permittivities (Supplementary Information). The relative energies associated to the anion binding process between the lowest-energy rotamer of protonated **1a** and the most favorable anion binding conformation (Fig. 2) were calculated and are shown in Table 1. Replacement of the pyrrole ring by a triazole heterocycle resulted in a lower binding energy for this compound compared to the related prodiginine analogs^30^. Nevertheless these compounds are well suited to interact with anions. In all cases the enthalpy difference follows the order bicarbonate >chloride > nitrate.

**Table 1:** Calculated binding energies (Kcal/mol) for compound **1a**.H^+^ and different anions. Data were calculated in media of different relative permittivities.

	Anion	Water ε = 80.1	DMSO ε = 46.7	Chloroform ε = 4.8	Gas Phase ε = 1
∆H	Cl^−^	−6.6	−7.5	−25.7	−101.0
	HCO_3_^−^	−11.7	−12.5	−30.7	−104.5
	NO_3_^−^	−6.4	−7.2	−24.1	−94.0

### Transmembrane transport

**Figure 3:**
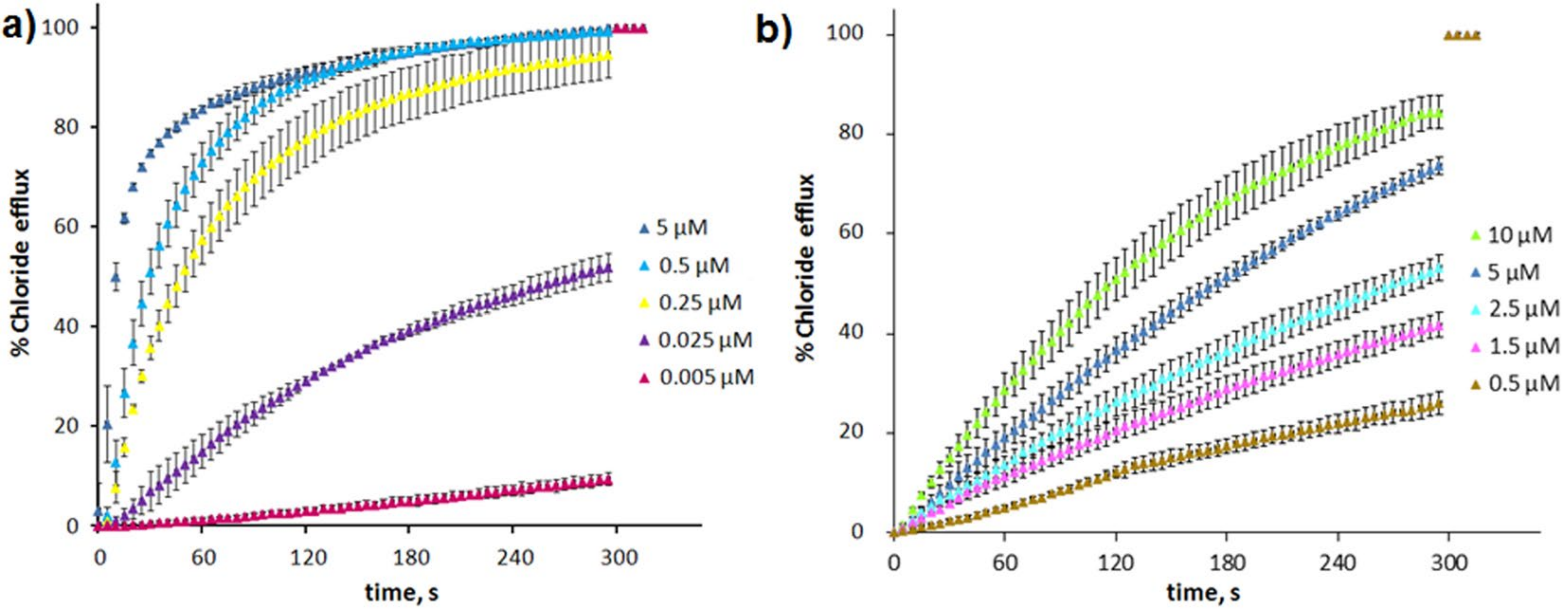
Chloride efflux upon addition of **1a**.HCl. (**a**) Time course of the normalized chloride concentration upon the application of the anion carrier at different concentrations, from 0.005 µM to 5 μM, as indicated in the figure. The LUV contained 489 mM NaCl and 5 mM phosphate buffer, pH 7.2, and were suspended in 489 mM NaNO_3_ and 5 mM phosphate buffer, pH 7.2. (**b**) The time course of the normalized chloride concentration upon the application of the anion carrier at different concentrations, from 0.5 µM to 10 μM, as indicated in the figure. The LUV contained 451 mM NaCl and 20 mM phosphate buffer, pH 7.2, and were suspended in 150 mM Na_2_SO_4_, 40 mM HCO_3_^−^ and 20 mM phosphate, pH 7.2. Each trace represents the average of at least three different experiments, carried out with at least three different batches of vesicles.

Chloride efflux experiments were carried out in large unilamellar vesicles (LUV) loaded with 489 mM NaCl, suspended in a 489 mM NaNO_3_ solution (pH 7.2). After equilibration of the chloride electrode (~60 s), a given concentration of the anion carrier was added, and the exponential change of the chloride concentration was monitored (see Fig. 3a). To measure the bicarbonate transport, LUVs were loaded with 451 mM NaCl and suspended over a 150 mM Na_2_SO_4_ solution (pH 7.2). After the electrode equilibration, the anion carrier was added, but not measurable chloride transport was observed, as the carrier is unable to exchange the sulfate anion with chloride. After 60 s, NaHCO_3_ was added to the external solution, to a final concentration of 40 mM, and the chloride efflux was measured with the electrode (Fig. 3b). To quantify the chloride transport, the initial value was set to zero chloride concentration and the time course was normalized to the final chloride concentration.

The activity of the anion carrier was evaluated measuring the activity observed after 300 s following the application of different concentrations of the compounds. The concentration to obtain half of the maximum effect, EC_50_, was estimated fitting the data with Hill’s equation. Data obtained for the four substances studied here, as well as the natural product prodigiosin, are shown in Table 2. This potency value is useful for comparative purposes, indicating the relative activity of the compounds. Prodigiosin was found to be the most active substance in all assays. This result is unsurprising since this natural product has been recognized among the most effective anionophores in the literature^5^. Nevertheless, the EC_50_ values of **1a**-**d** are remarkably low, and these compounds proved extremely active in the chloride/nitrate transport assay. Significant differences were observed when the external anion is a mixture of bicarbonate and sulfate. The calculated EC_50_ values are usually higher in this assay compared to the chloride/nitrate transport experiments. On the other hand, the differences between EC_50_ values depending on whether the external anion was nitrate or bicarbonate were much larger in the case of compounds **1a**-**d**. This result evidences the difference in the binding cleft of these two types of anionophores and indicates that **1a**-**d** are less efficient carriers in this assay compared to prodigiosin. The calculated Hill’s coefficients are consistent with the 1:1 stoichiometry observed both in solution and the solid state.

**Table 2:** Anion transmembrane transport facilitated by **1a**-**d** and prodigiosin.

Compound	NO_3_^−^/Cl^− (a)^		HCO_3_^−^/Cl^− (b)^		Initial quenching rate^(c)^ (s^−1^)
	EC_50_ (nM)	Hill’s coefficient	EC_50_ (nM)	Hill’s coefficient	
**1a**	24	1.39	1911	0.94	0.009
**1b**	30	1.27	2609	0.72	0.008
**1c**	18	1.17	1617	0.65	0.020
**1d**	50	1.43	42783	0.63	0.007
prodigiosin	2	1.13	58	0.91	0.079

^a^LUV loaded with 489 mM NaCl dispersed in 489 mM NaNO_3_ (5 mM phosphate buffer, pH 7.2). ^b^LUV loaded with 451 mM NaCl dispersed in 150 mM Na_2_SO_4_ (20 mM phosphate buffer, pH 7.2) upon addition of a NaHCO_3_ pulse to make the external bicarbonate concentration 40 mM. ^c^LUV loaded with 102.2 mM NaNO_3_ dispersed in 102.2 mM NaNO_3_ (20 mM phosphate buffer, pH 7.2) upon addition of a NaCl pulse to make the external chloride concentration 10 mM.

**Figure 4:**
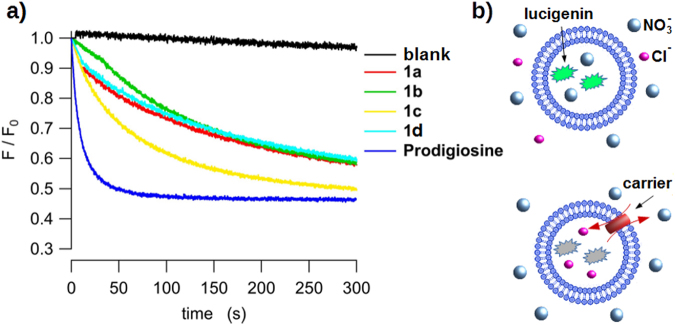
Chloride influx measurements. (**a**) Chloride influx measured upon addition of **1a**-**d** and prodigiosine measured with the chloride probe lucigenin. LUV containing lucigenin in NaNO_3_ were suspended in an isomolar NaNO_3_ solution. NaCl was added to the external solution at the beginning of the experiment. Black trace: **1a**.HCl, green trace: **1b**.HCl, red trace: **1 c**.HCl, blue trace: **1d**.HCl, magenta trace: prodigiosin and brown trace: blank (10 µL MeOH). Compound concentrations 50 nM. (**b**) Cartoon representation of lucigenin assay.

The chloride transport ability of **1a**-**d** was further explored using a fluorescence based assay in which lucigenin, a fluorescent probe which emission is quenched by halides, is encapsulated in nitrate containing vesicles^31^. Internalization of chloride is thus signaled by the quenching of the fluorescence. This assay allows directly monitoring of chloride influx using moderate chloride gradients (Fig. 4a). The time course of the fluorescence *I*(t) can be fitted with Equation 1:

1 $$\frac{I(t)}{I(0)}=(1-{I}_{max})+{I}_{max}{e}^{-t/\tau }$$

where *I*(0) is the fluorescence at zero time, *I*_*max*_ is the asymptotic fluorescence intensity, corresponding to the maximum quenching and τ is the relaxation constant of the process. The initial quenching rate, representing the flux at t = 0, equals to 1/τ (Table 2). The results obtained measuring the chloride influx with a fluorescent probe are in good agreement with those obtained measuring the chloride efflux with the chloride selective electrode, being **1c** the most active compound among **1a-d** and prodigiosin the most active anion carrier.

When performing transport experiments it became evident that the composition of the external solution greatly impacted in the observed chloride efflux. In fact, almost negligible chloride efflux was observed when the external solution contains only highly hydrophilic anions such as sulfate or gluconate. These observations support the hypothesis that the presence of an external permeable anion is necessary to sustain the anionophore-chloride efflux in LUV to equilibrate the internal and external chloride concentration. This could be due to an unidirectional charge movement (chloride moving from inside to outside the LUV), without another ion movement balancing the charge distribution at the sides of the bilayer. Thus, the creation of an electrochemical gradient arrests the chloride efflux. The polarization of the membrane under these conditions was proved in a fluorescence assay using the membrane potential sensitive dye safranin O (Supplementary Fig. 23).

**Figure 5:**
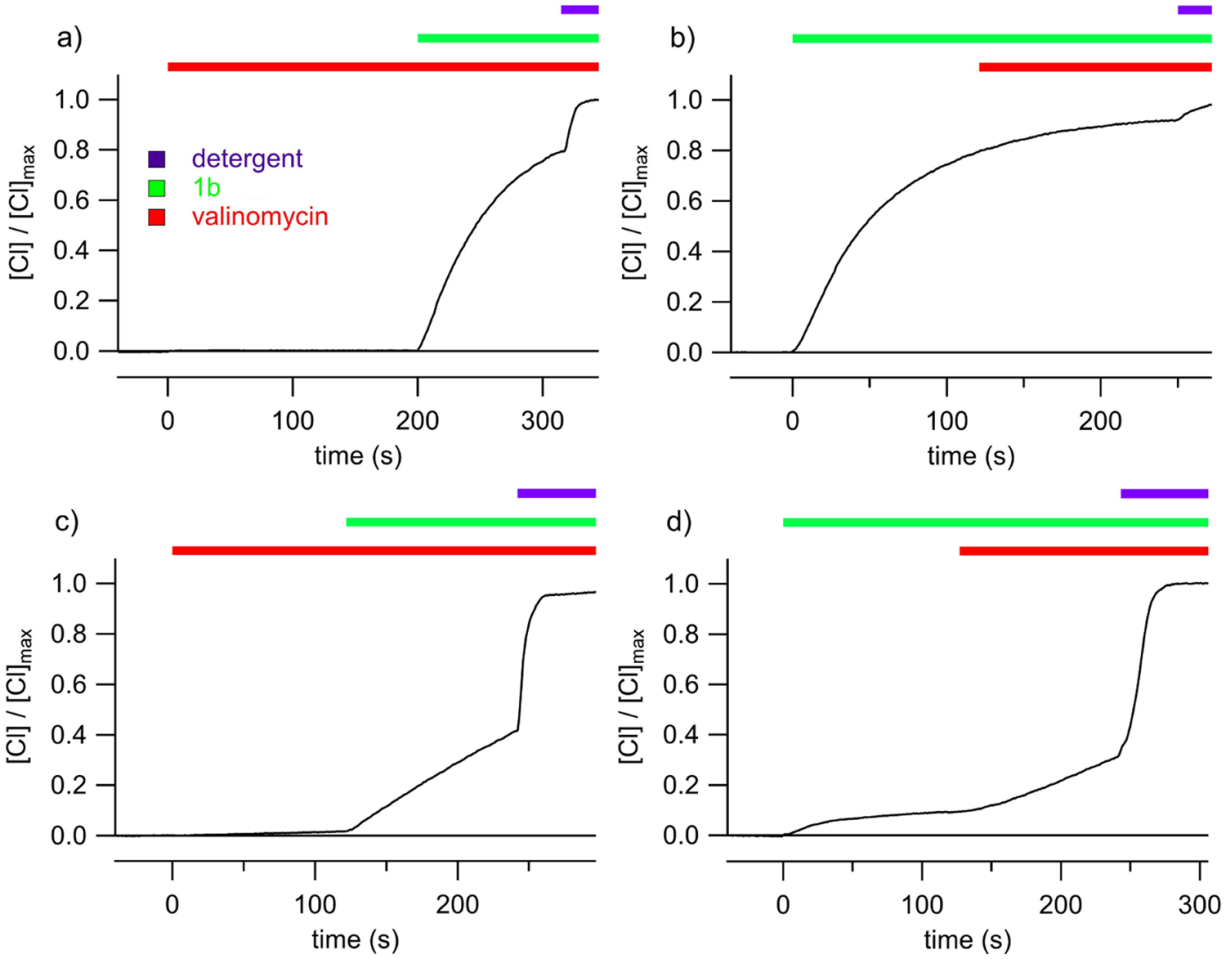
Chloride transport in valinomycin-“shunted” membranes. Chloride efflux induced by the anionophore **1b** (4 µM) recorded in LUVs with 450 mM KNO_3_ and 20 mM HEPES, pH 7.0 in the external solution (**a** and **b**), and with 300 mM K_2_SO_4_ and 20 mM HEPES, pH 7.0 in the external solution (**c** and **d)**. The LUV contained 450 mM KCl and 20 mM HEPES, pH 7.0. (**a**) Addition of valinomycin (1 µg/ml) does not induce any chloride efflux, and successive application of the anionophore induced a consistent chloride efflux. (**b**) When valinomycin is added after **1b** induced chloride efflux is initiated, it does not modify the time course of the transport. (**c**) In the presence of the external impermeable anion, a robust chloride efflux is observed when the anionophore **1b** is applied in presence of valinomycin. (**d**) The anionophore elicits a very small chloride efflux when the external anion is impermeable, but it significantly increases upon the application of valinomycin.

To further prove this mechanism, we performed series of experiments using the potassium selective carrier valinomycin in the presence of potassium salts to “shunt” the membrane potential. First, we explored the assay in which nitrate was present in the external solution. Under these conditions, valinomycin alone did not induce any chloride efflux, but addition of **1b** resulted in the expected chloride efflux (Fig. 5a). Accordingly, addition of valinomycin did not modify the ion sensitive electrode record in an assay carried out adding the anionophore first (Fig. 5b). When the same protocols are applied to LUVs using sulfate (an impermeable anion) as external anion, the action of either valinomycin or **1b** alone did not result in significant chloride efflux. On the other hand, **1b** induced a significant efflux in the presence of valinomycin (Fig. 5c). Similarly, the small anionophore-induced chloride efflux recorded in external sulfate is significantly increased upon the application of valinomycin (Fig. 5d). These results are consistent with a potassium and chloride transport facilitated by the concerted action of valinomycin and **1b**, preventing the building up of a membrane potential and thus facilitating the observed chloride efflux.

### Anion transport in living cells

**Figure 6:**
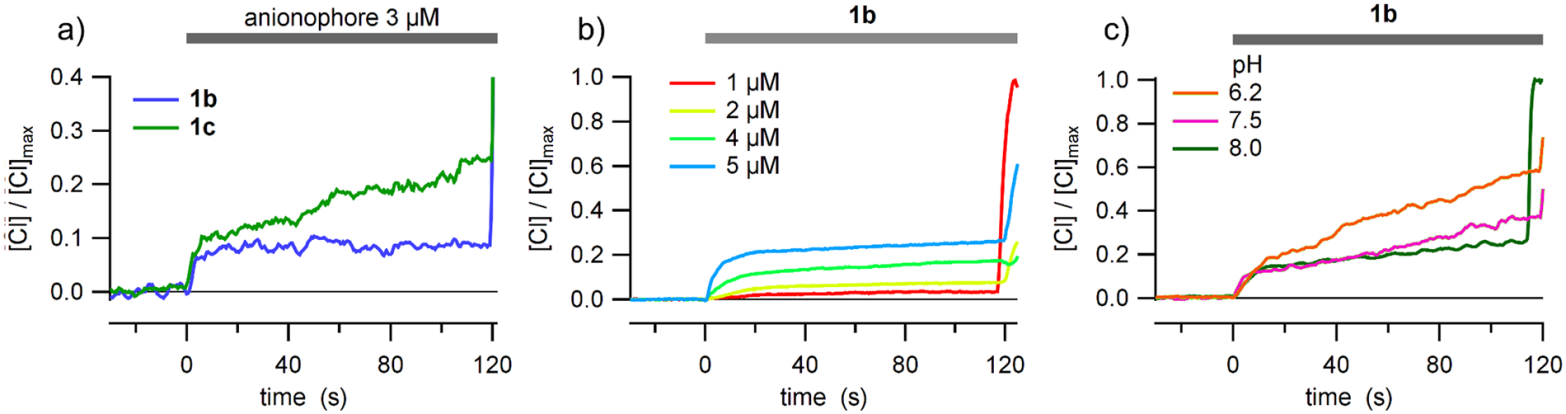
Anionophore-induced chloride efflux in HEK-293 cells. (**a**) Chloride efflux evoked by the application of 3 µM of **1b** and **1c**, as indicated in the graphic. (**b**) Chloride efflux upon application of different concentrations of **1b**. (**c**) Anionophore-induced chloride efflux measured at different extracellular pH, as indicated in the figure. Extracellular solution was 136 mM NaNO_3_, 3 mM KNO_3_, 2 mM Ca(NO_3_)_2_, 20 mM HEPES, 11 mM Glucose, pH 7.4, except for (**c**), where pH was adjusted as indicated.

We explored the ability of compounds **1a-d** to facilitate anion transport in living cells. Thus, chloride efflux from HEK-293 cells suspended in a chloride free medium was measured using a chloride selective electrode. Figure 6a shows records of chloride efflux measured in HEK-293 cells, after application of 3 µM **1b** or **1c**. The anionophore induces a fast chloride efflux, initial rate (0.041 s^−1^ and 0.029 s^−1^ for **1b** or **1c** respectively), followed by a slower chloride efflux. The first, fast, component is related with the anionophore-driven chloride transport, whereas the second component is variable and depends on the cell batch. We concluded that the second component of the chloride efflux has information about the cellular regulation of chloride and other anions, and needs further study to be well understood.

We also measured the chloride efflux at different concentrations of **1b** (Fig. 6b). The chloride efflux was found to be proportional to the concentration of anionophore. Interestingly, the effect of the anionophores also depends on the extracellular pH. In Fig. 6c is shown that the efficacy of transport of **1b** increases as extracellular pH is more acid.

**Figure 7:**
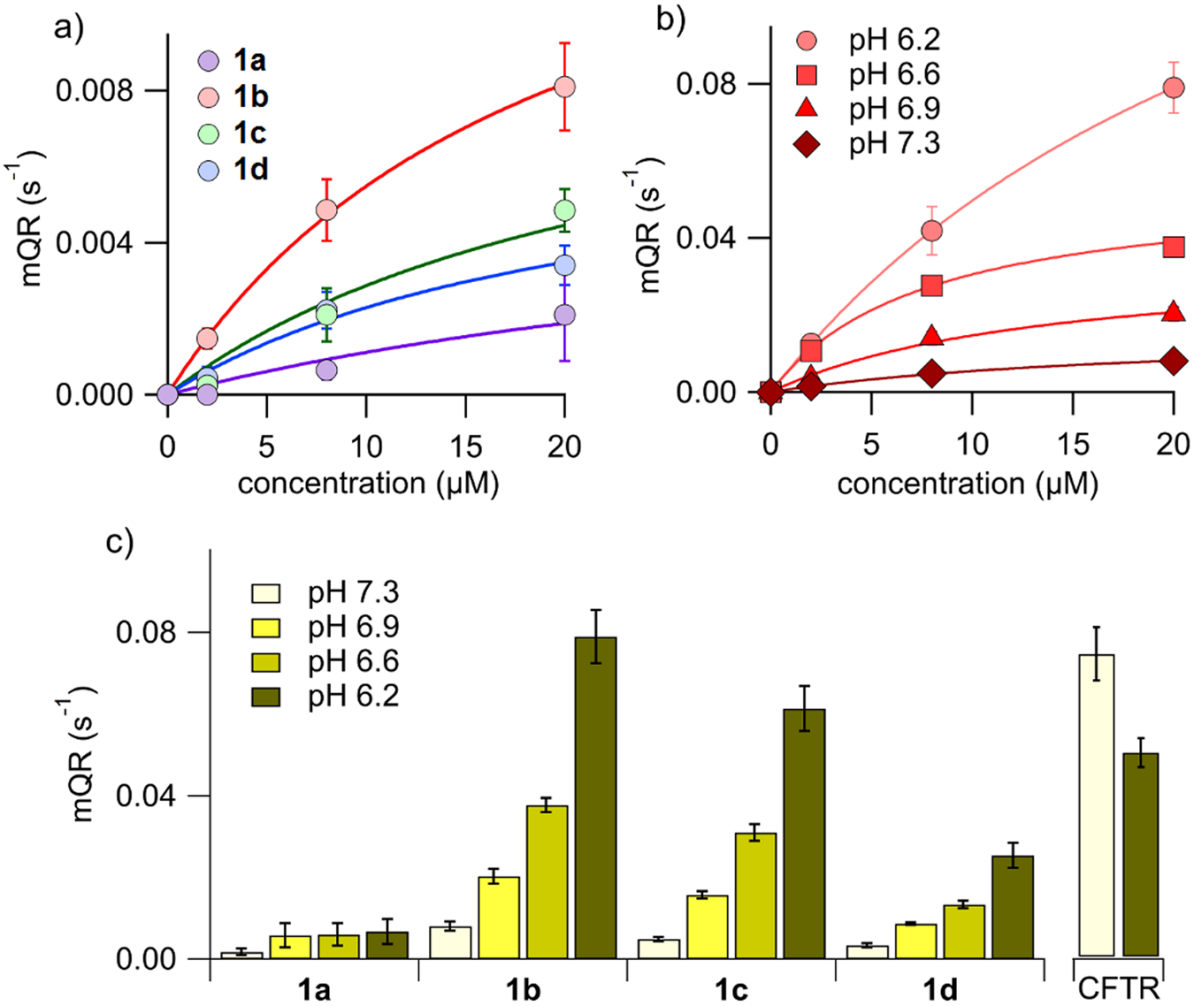
Iodide influx measurements in FTR cells. (**a**) Dose-response relationship of compounds **1a**-**d**. FTR-cells expressing the iodide-sensitive YFP protein were incubated in phosphate-buffered solution containing 137 mM NaCl. Iodide influx was estimated by the YFP fluorescence quenching upon addition of ICl to a final concentration of 100 mM. The maximum fluorescence quenching rate at each concentration is plotted against the carrier concentration. Each symbol is the mean of 6–10 different experiments and vertical bars are the standard error of the mean. The continuous lines are the fit of mean points to Langmuir model. (**b**) The activity of compound **1b** was determined as function of concentration when the extracellular NaI solution was buffered at different pH values, from 7.3 to 6.2. (**c**) The activity of compounds **1a**-**d** depended on extracellular pH. The figure depicted the maximum quenching rate of the four compounds at a concentration of 20 µM at the indicated extracellular pH values. Each value is the mean of 4–10 experiments. As comparison, the activity of wild type CFTR at two different extracellular pH values is shown (n = 4). CFTR was maximally activated by applying 20 µM forskolin, and a CFTR potentiator compound (10 µM genistein) before starting the fluorescence assay.

These efflux measurements proved difficult because cells regulate the intracellular chloride maintaining it low (chloride concentration inside epithelium cells ≤30 mM) thus, upon removing the extracellular chloride, beside the alteration of the membrane potential that will alter the cell homeostasis, the effective gradient is 15-fold smaller than that used in LUV experiments, and therefore the chloride efflux must be proportionally smaller. Differently, in the measurements of iodide influx, is possible to measure the anion transport with a significant gradient. The time course of the decay of the YFP fluorescence in FRT cells, upon addition of NaI, was measured after treatment with compounds **1a**-**d** at different concentrations. After background subtraction and normalization for the maximal value before NaI addition, the signal decay was fitted with a double exponential function and the maximum rate of fluorescence decay (maximum quenching rate, mQR) was derived. This parameter is a direct indication of the activity of the tested compound. The mQR at each concentration permitted to construct a dose-response relationship for each compound. Fitting the data to a Langmuir model the apparent EC_50_ values were calculated at >40 µM, 17.1 ± 1.3 µM, 21.8 ± 2.7 µM and 24.9 ± 3.3 µM for compounds **1a**, **1b**, **1c** and **1d**, respectively. The larger mQR and the lower value of EC_50_ were observed for **1b** (Fig. 7a).

As observed measuring the carrier-driven chloride efflux, the activity of compounds **1a-d** depends on the extracellular pH. A significant increase in the mQR was observed when the extracellular pH was set at slightly acidic values, up to pH = 6.2 (Fig. 7c). The activity of wild type CFTR, measured by incubating FRT cells with forskolin, an agonist that increases the intracellular cAMP, plus the CFTR potentiator genistein, was also evaluated for comparison purposes. These measurements were carried out at pH 7.3 and 6.2 (Fig. 7c). Remarkably, this is the opposite trend observed for activated wild type CFTR, and, under these conditions, compound **1b** at 20 µM showed comparable activity to the measured activity for CFTR.

**Figure 8:**
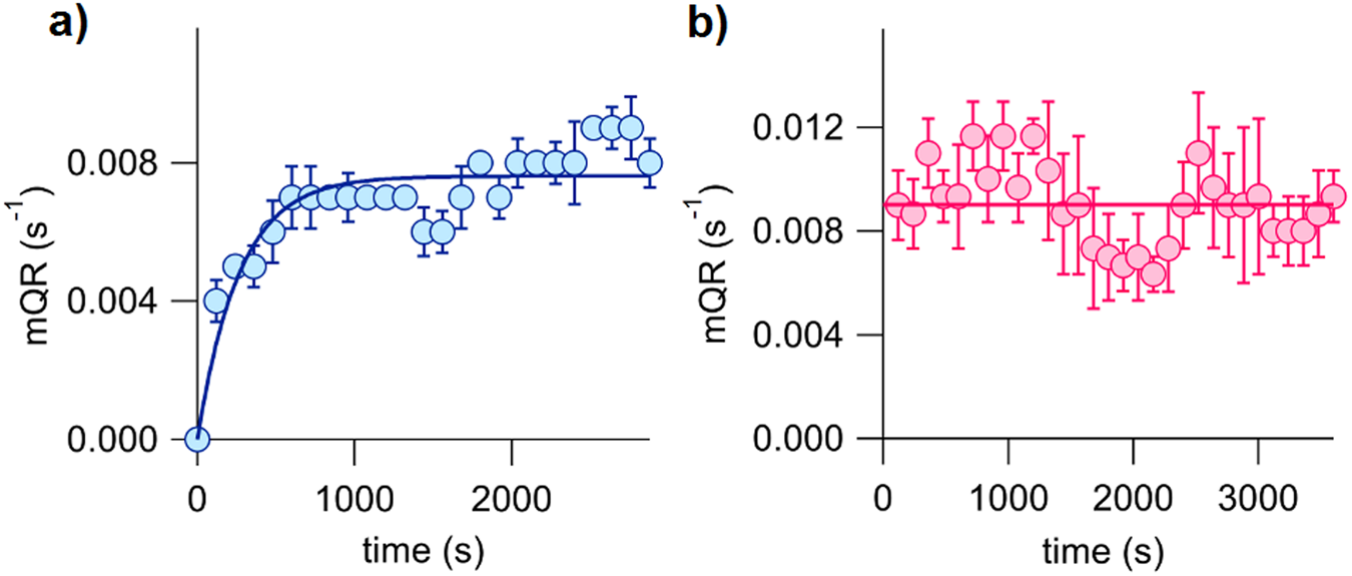
Time course of the incorporation and the stability of compound **1b** in the plasma membrane. FTR-cells expressing the iodide-sensitive YFP protein were incubated in phosphate-buffered solution containing 137 mM NaCl. Iodide influx was estimated by the YFP fluorescence quenching upon addition of ICl to a final concentration of 100 mM. (**a**) Kinetics of the incorporation of compound **1b** in the plasma membrane. The activity of the compound (at 12 µM concentration) was measured, as maximum quenching rate, as function of time. (**b**) Stability of compound **1b** in the plasma membrane. After 45 min incubation with 12 µM **1b**, cells were washed twice and the residual transport activity measured during 60 minutes. Symbols are means of 3 different experiments and vertical bars are standard error of the mean.

To investigate the time course of incorporation and the stability of this class of compounds in the plasma membrane, we selected compound **1b** because of its higher activity, which allows to easily measure the anion transport. For determining the incorporation in the plasma membrane, compound **1b** was added to the cells seeded in a multiwell plate, and the fluorescence assay was recorded in a different well every two minutes. The data obtained indicated that the incorporation of **1b** is fast. Two minutes after the compound addition the activity of the **1b** was found to be about 50% of the final activity (Fig. 8a). The transport rate continuously increased in the following minutes reaching the 95% of maximum incorporation in 12.9 minutes (time constant 4.3 ± 0.4 min). In order to elicit a useful action, the compounds should be able to reside in the plasma membrane a reasonable amount of time. To explore this, FRT cells were incubated for 45 minutes with compound **1b** and then the cells were washed twice with phosphate buffer solution to remove the compound. The fluorescence was then recorded every two minutes (Fig. 8b). The transport rate remained substantially unchanged within one hour, being the decrease of the maximum quenching rate over this period not statistically significant. Thus we can conclude that compound **1b** is able to facilitate sustained iodide transport activity for significant periods of time.

### Cellular effects of the compounds

An evaluation of the toxicity of the compounds revealed that the viabilities in cell lines examined were not affected or slightly decreased for treatment with 5 µM of **1a**-**d**, and in some cases even at 20 μM; conversely, high cytotoxicity was observed for prodigiosin in the same assays (Supplementary Figs 24 to 26).

We also investigate the impact of these compounds on the intracellular pH^32,33^. We measured of changes in the intracellular pH with the fluorescent dye SNARF-1 in A549 cells upon treatment with 10 µM of **1a**-**d**. The results indicated very limited changes in the pH level (Supplementary Fig. 27). Moreover, investigating potential changes in acidic compartments of the cell using the vital staining acridine orange, we observed that, in A549 cells, the number of orange particles, correlating with acidic organelles, is not significantly changed by the treatment with 10 µM of **1a**-**d** (Supplementary Fig. 28). This result evidenced the inability of **1a-d** to deacidify acidic organelles such as lysosomes.

## Discussion

We have presented a series of compounds inspired in the structure of natural product prodigiosin. These compounds are the result of replacing one of the pyrrole groups characteristic of the natural derivatives by a 1,2,3-triazole heterocycle. The 1,2,3-triazole display a polarized C-H group which is an effective hydrogen bond donor. As demonstrated from NMR experiments and the crystalline structure of the compounds, they have the ability to bind anions through the hydrogen bonding cleft defined by the two pyrrole N-H and the triazole C-H groups. This concept has been corroborated from ab initio electronic structure calculations, which also suggested that the most favorable binding conformation is the same as the one characterized in the solid state. Thus these compounds present a similar hydrogen bonding cleft to natural prodiginines. As model derivatives we selected alkyl-substituted triazoles, so the overall lipophilicity of the molecule can be explored and tuned to maximize de transport properties of these derivatives. Compounds **1a-d** are able of exchanging chloride with nitrate or bicarbonate across the bilayer membrane in a vesicle models (Fig. 3). However, these carriers are unable to transport highly hydrophilic anions such as sulfate or gluconate. The presence of these impermeable anions results in the building up of a transmembrane potential difference (Supplementary Fig. 23), that impedes further chloride to be carried, virtually blocking the anion transport process. This hypothesis was verified by dissipating the potential difference upon addition of valinomicyn, a potassium carrier that would “shunt” the lipid bilayer. This results in chloride transport, even in the presence of an impermeable anion in the opposite side (Fig. 5). A corollary of this observation is that these carriers do not need to exchange anions to carry out their transport tasks, but such exchange is just limited to the maintenance of electroneutrality across the lipid bilayer.

In stark contrast to other anion carriers such as prodigiosin, compounds **1a-d** display very limited toxicity, opening the possibility of using these substances as minimalistic surrogates for replacing transmembrane transport function or in “channel replacement” therapies. CF hallmark is the lack or dysfunction of CFTR, resulting in an impaired transport of chloride and bicarbonate in the apical membrane of epithelia, with catastrophic consequences on the health of the affected subjects. The capacity of these substances of carrying both, chloride and bicarbonate, configures compounds **1a-d** as potentially good candidates for cystic fibrosis therapy. Moreover, this strategy constitutes a mutation independent approach, addressing the root problem of CF patients, independently of the genetic mutation responsible for their condition.

To back up this proposal, we have performed a series of experiments to evaluate whether compounds **1a-d** are able to transport anions in cells. Therefore, we have carried out a series of experiments measuring the iodide influx and the chloride efflux in living cells, providing a proof of concept for the possibility to use these substances to induce anion transport in an epithelia. Indeed, the anion transport capacity observed in FRT cells is of the same order of magnitude of the CFTR response in the same preparation. However, it should be noted that the activity of the wild type CFTR measured as short-circuit current on FRT epithelia stably transfected with CFTR is much higher (10.6 times) than CFTR endogenously expressed in primary human bronchial epithelia. Thus, it can be anticipated than the mQR measured in the described assay would be significantly higher as well. Thus it is likely that much lower concentrations of the compounds **1a-d** could display comparable transport activities to endogenous wild type CFTR in bronchial epithelia.

An interesting observation is that these compounds are more efficient when extracellular pH is acid (see Figs 6 and 7). This result could be explained because at acidic pH a larger fraction of the anionophore is protonated, increasing the ionophoric activity (calculated pKa of **1a**-**d** vary from 6.22 to 6.43). This result bodes well again for the potential efficacy of these compounds, since the lung epithelia of CF patients is known to be more acidic than normal^34,35^. The analysis of anionophore-induced anion transport in cells needs, in any case, to be extended, and perhaps the measurement methods have to be refined to identify the best suited compounds to become drug candidates for therapy.

These experiments demonstrate that these anionophores have a limited toxicity, and could be used to promote anion transport in cells, i.e., are good candidates to replace the defective or missing CFTR in an attempt to design a new, mutant independent cystic fibrosis therapeutic approach. This proof of concept represents an encouraging premise for future development towards a mutant-independent cystic fibrosis therapy.

## Methods

### Synthesis of compounds

Briefly, compounds **1a**-**d** (Fig. 1) were prepared by acid-catalyzed condensation of 3-methoxy-5-(1-alkyl-1H-1,2,3-triazol-4-yl)-1*H*-pyrrole-2-carbaldehyde and 2,4-dimethyl-1*H*-pyrrole or 2-methyl-3-pentyl-1*H*-pyrrole. The precursor aldehyde was prepared by standard click chemistry reaction between 1-azidobutane or 1-azidooctane and the corresponding 5-ethynylpyrrole carbaldehyde. Full experimental procedures are given in the Supplementary Information. These compounds are inspired in the structure of natural products prodiginines, replacing one of the pyrrole groups by a 1,2,3-triazole moiety. A detailed description of the synthesis and the chemical characterization of the compounds are described in the Supplementary Results.

### Anion transport in unilamellar vesicles

The activity of these compounds as transmembrane carriers was assayed in model phospholipid large unilamellar vesicles (LUVs) with average diameters of 100 and 200 nm. LUVs were prepared by extrusion across polycarbonate membranes, resulting in good control of the vesicle size distributions and the composition of encapsulated and external aqueous phases. The chloride efflux from chloride-loaded vesicles was monitored recording the time course of the external chloride concentration using ion selective electrodes. Release of all encapsulated chloride by addition of detergent allows the normalization of the data. Influx of chloride was measured encapsulating the chloride fluorescent probe lucigenin. Polarisation of the LUV bilayers was evaluated with Safranin O. A detailed description of the measurement methods are described in the Supplementary Information.

### Cell cultures

Human lung adenocarcinoma (A549), human mammary adenocarcinoma (MCF-7), human mammary epithelial (MCF-10A), Fischer rat thyroid (FRT) and human embryonic kidney (HEK-293) cell lines were grown in standard conditions as described in Supplementary Information. Cell viability was evaluated with the MTT assay on A549, MCF-7 and MCF-10A cells. Intracellular pH was measured in A549 cells by cytometry using a ratiometric fluorophore SNARF-1. Acidification of intracellular compartments was verified with the acridine orange assay. Detailed protocols for the assays are described in Supplementary Information.

### Anion transport assays in living cells

The iodide influx was measured in FRT cells stably transfected with a halide-sensitive yellow fluorescence protein (YFP-H148Q/I152L)^36,37^. This YFP variant has a higher affinity for iodide than for chloride, allowing to detect iodide concentration changes without significant interferences from small chloride changes^36–38^. Cells, pre-treated with the anion carrier, were perfused with iodide, and the quenching of the fluorescence represents anion influx.

The chloride efflux was measured in HEK-293 cells suspended in a chloride-free solution. After an initial equilibration, chloride efflux was induced by a small volume (<1%) of ionophore, and the concentration of chloride in the extracellular solution was monitored with a chloride-sensitive electrode. The measurement was concluded with the addition of the sodium dodecyl sulfate (SDS) to break off the membranes and measure the total chloride content in the cells. A detailed description of the measurement of anion fluxes in living cells are described in the Supplementary Information.

## Electronic supplementary material

Supplementary Information
