# Supplementary material for: Small molecule anionophores promote transmembrane anion permeation matching CFTR activity
Source: Sci Rep. 2018 Feb 8;8:2608. doi: 10.1038/s41598-018-20708-3 (PMC5805763; doi:10.1038/s41598-018-20708-3)
Supplement: Supplementary file 1 — Supplementary Information [file 41598_2018_20708_MOESM1_ESM.pdf]

Supplementary Information

Small molecule anionophores promote transmembrane anion permeation matching CFTR activity

Elsa Hernando[1,5], Valeria Capurro[2,5], Claudia Cossu[3,5], Michele Fiore[3],
María García-Valverde[1], Vanessa Soto-Cerrato[4], Ricardo Pérez-Tomás[4], Oscar Moran[3*],
Olga Zegarra-Moran[2], and Roberto Quesada[1*]

[1]Departamento de Química, Universidad de Burgos, 09001 Burgos, Spain

[2]U.O.C. Genetica Medica, Instituto Giannina Gaslini, Genoa, Italy

[3]Istituto di Biofisica, CNR, Genoa, Italy

[4]Cancer Cell Biology Research Group, Department of Pathology and Experimental Therapeutics, Faculty of Medicine, University of Barcelona, Barcelona, Spain

[5]These authors contributed equally to this work.

Corresponding Authors:

* rquesada@ubu.es

* oscar.moran@cnr.it

SUPPLEMENTARY METHODS

Chemical synthesis

General. Commercial reagents were obtained from commercial suppliers and used without any further purification. All reactions involving air-sensitive compounds were assembled under a N_2 atmosphere in oven-dried glassware with magnetic stirring. Thin layer chromatography (TLC) was performed on Al foils coated with silica gel 60 with F_{254} indicator, and Al foils coated with aluminium oxide 60 with F_{254} indicator, neutral. The chromatograms were visualized by UV light (254 nm and 366 nm). NMR spectra were recorded in Varian Mercury-300 MHz and Varian Unity Inova-400 MHz spectrometers. Chemical shifts (δ) are reported in parts per million (ppm) and calibrated using the residual solvent peak as reference, coupling constants are reported in Hz. Data are reported as follows: chemical shift, multiplicity (splitting pattern abbreviations are: s: singlet, br s: broad singlet, d: doublet, t: triplet, q: quartet, m: multiplet). High resolution mass spectra (HRMS) were recorded on a Micromass Autospec S-2 spectrometer using EI at 70eV.

General procedure for the synthesis of 3-methoxy-5-(1*H*-1,2,3-triazol-4-yl)-1*H*-pyrrole-2-carbaldehydes 2: Commercially available 4-methoxy-3-pyrrolin-2-one was subjected to a Vilsmeier-Haack reaction using $POBr_3$ and diethylformamide in DCM[1]. A mixture of the obtained azafulvene (1 equiv), ethynyltrimethylsilane (2 equiv), $PdCl_2(PPh_3)_2$ (0.02 equiv.), CuI (0.04 equiv.) in Et_2NH:Et_3N (1:1) (6 mL/mmol) was stirred under a nitrogen atmosphere at 80 ºC until complete consumption of starting material, as monitored by TLC (Hexane:AcOEt 2:1). Once cooled, the reaction was diluted with water (20 mL/mmol) and extracted with CH_2Cl_2 (2 × 30 mL). The combined organic layers were dried over anhydrous Na_2SO_4 and concentrated at reduced pressure to give the required compound **4** in 66 % yield. This last compound was treated with TBAF (1 equiv, 1 M solution in THF) in THF as solvent (6 mL/mmol) at rt for 3 h. The reaction was diluted with dichloromethane and washed (3 x 20 mL) with water. The organic layer was passed over 2 cm of silica gel pad with a filter plate (pore 2) using AcOEt, furnishing compound **5** (70%).

Azides **3** were synthesized from the corresponding aliphatic halide (Scheme S1) and NaN_3. In a round bottom flask the halide was added over a mixture of water and dioxane (7:3) (2 mL/mmol), after that the pulverized NaN_3 (1.5–3 equiv.) was incorporated to the solution and stirred vigorously at 100 ^{o}C. The reaction was monitored by ^{1}H NMR. Once finished, the flask was placed in an ice-water bath, water was added (10 mL/mmol) and it was left stirring for 10 minutes. The reaction was then extracted with ether (3 x 60 mL). The organic layer was washed once with brine, dried over anhydrous Na_2SO_4 and concentrated at reduced pressure to give the corresponding azides as colorless oils. Butylazide was stored as concentrated solutions at -20ºC.

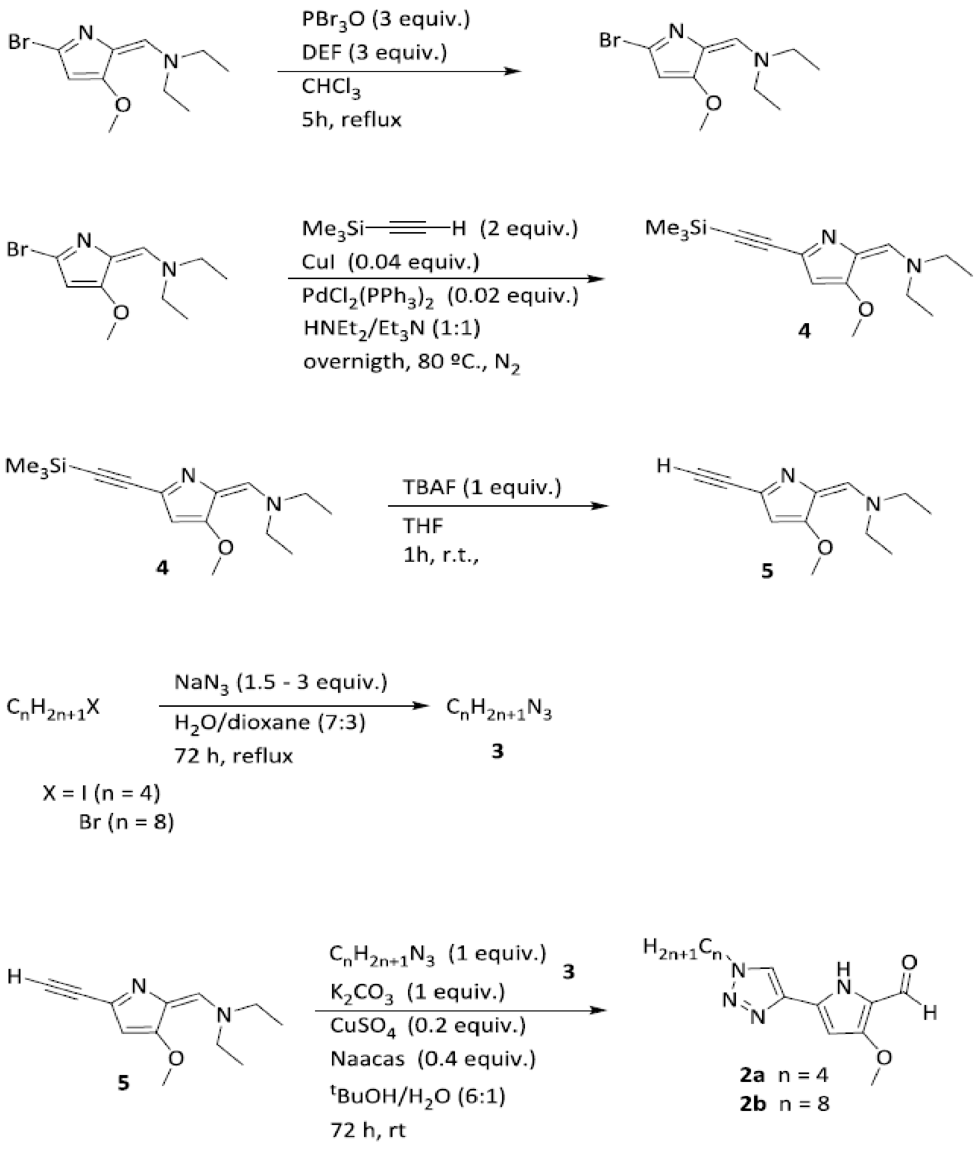

Supplementary Figure 1. Syntheses of aldehydes **2a,b**.

Using a CuAAC reaction, $CuSO_4$ (0.2 equiv.) dissolved in water (0.35 mL) was added to a mixture of the alkyne **5** (1 mmol) and the corresponding azide **3** (1 equiv.), followed by ^tBuOH (6.6 mL), K_2CO_3 (1 equiv.), sodium ascorbate (0.4 equiv.) and water (0.35 mL). The heterogeneous mixture was stirred vigorously until TLC (CH_2Cl_2/AcOEt 3:1) analysis indicated complete consumption of reactants (1-3 days). The reaction mixture was diluted with 50 mL of NH_4OH, after 30 minutes it was extracted with Et_2O (3 x 25 mL). The combined organic layers were washed with brine (1 x 40 mL), dried over anhydrous Na_2SO_4 and concentrated at reduced pressure. The obtained solid was purified by column chromatography on silica gel (CH_2Cl_2/AcOEt 3:1) to yield the corresponding aldehyde **2**.

The spectroscopic and analytical data for the new aldehydes and their precursors are reported below:

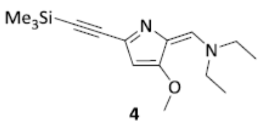

4

N-ethyl-N-((3-methoxy-5-((trimethylsilyl)ethynyl)-2*H*-pyrrol-2-ylidene)methyl) ethanamine (4). Yield: 66 %. ^{1}H NMR (300 MHz, $CDCl_3$): δ = 7.09 (s, 1H), 5.78 (s, 1H), 4.24 (q, J = 7.1 Hz, 2H), 3.73 (s, 3H, OCH_3), 3.40 (q, J = 7.2 Hz, 2H), 1.31–1.23 (m, 6H), 0.21 (s, 9H); ^{13}C NMR (75 MHz, $CDCl_3$): δ = 164.79 (C), 140.61 (CH), 139.03 (C), 122.35 (C), 103.94 (C), 98.31 (CH), 94.32 (C), 57.89 (OCH_3), 51.08 (CH_2), 44.80 (CH_2), 14.63 (CH_3), 12.68 (CH_3), 0.13 (3 CH_3). HRMS (EI) m/z $[M]^+$ calc for $[C_{15}H_{24}N_2OSi]$ 276.1658; found: 276.1655.

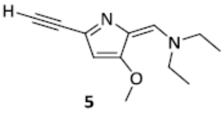

5

N-ethyl-N-((5-ethynyl-3-methoxy-2*H*-pyrrol-2-ylidene)methyl)ethanamine (5). Yield: 70%. ^{1}H NMR (300 MHz, $CDCl_3$): δ = 7.12 (s, 1H), 5.78 (s, 1H), 4.23 (q, J = 7.2 Hz, 2H), 3.74 (s, 3H, OCH_3), 3.42 (q, J = 7.2 Hz, 2H), 3.10 (s, 1H), 1.31–1.25 (m, 6H). ^{13}C NMR (75 MHz, $CDCl_3$): δ = 164.74 (C), 140.82 (CH), 137.92 (C), 122.09 (C), 97.84 (CH), 82.79 (C), 76.91 (CH), 57.89 (OCH_3), 51.28 (CH_2), 44.86 (CH_2), 14.61 (CH_3), 12.60 (CH_3). HRMS (EI) m/z $[M]^+$ calcd for $[C_{12}H_{16}N_2O]$ 204.1263; found: 204.1267. HRMS (EI) $[M]^+$ m/z calc for $[C_{12}H_{16}N_2O]$ 204.1263; found: 204.1267.

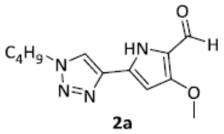

2a

5-(1-butyl-1H-1,2,3-triazol-4-yl)-3-methoxy-1*H*-pyrrole-2-carbaldehyde (2a). Yield: 64%. ^{1}H NMR (300 MHz, $CDCl_3$): δ = 10.95 (br s, NH, 1H), 9.51 (s, CHO, 1H), 8.34 (s, 1H), 6.40 (s, 1H), 4.42 (t, J = 7.1 Hz, 2H), 3.93 (s, 3H, OCH_3), 1.98–1.88 (m, 2H), 1.46–1.33 (m, 2H), 0.98 (t, J = 7.3 Hz, 3H); ^{13}C NMR (75 MHz, $CDCl_3$): δ = 174.44 (CHO), 159.79 (C), 140.01 (C), 131.72 (C), 121.25 (CH), 119.10 (C), 93.55 (CH), 58.23 (OCH_3), 50.42 (CH_2), 32.31 (CH_2), 19.80 (CH_2), 13.58 (CH_3); HRMS (EI) m/z $[M]^+$ calc for $[C_{12}H_{16}N_4O_2]$ 248.1273; found: 248.1282.

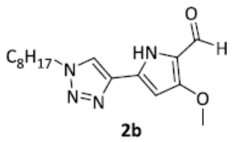

2b

3-methoxy-5-(1-octyl-1H-1,2,3-triazol-4-yl)-1*H*-pyrrole-2-carbaldehyde (2b). Yield: 60 %. ^{1}H NMR (300 MHz, $CDCl_3$): δ = 11.20 (br s, NH, 1H), 9.48 (s, CHO, 1H), 8.43 (s, 1H), 6.44 (s, 1H), 4.40 (t, *J* = 7.2 Hz, 2H), 3.92 (s, 3H, OCH_3), 1.95–1.91 (m, 2H), 1.33–1.24 (m, 10H), 0.86 (t, *J* = 6.1 Hz, 3H); ^{13}C NMR (75 MHz, $CDCl_3$): δ = 174.31 (CHO), 160.04 (C), 140.02 (C), 132.12 (C), 121.42 (CH), 119.13 (C), 93.56 (CH), 58.19 (OCH_3), 50.69 (CH_2), 31.83 (CH_2), 30.34 (CH_2), 29.17 (CH_2), 29.08 (CH_2), 26.57 (CH_2), 22.71 (CH_2), 14.18 (CH_3). HRMS (EI) m/z $[M]^+$ calcd for $[C_{16}H_{24}N_4O_2]$ 304.1899; found: 304.1896.

General procedure for the synthesis of (1*H*-1,2,3-triazol-4-yl)-2-((1*H*-pyrrol-2-yl))-3-methoxy-2*H*-pyrrol-1-ium chloride 1: Under a nitrogen atmosphere and at r.t., 5 mL of degassed MeOH were added over the corresponding carbaldehyde **2** (0.3 mmol) and the corresponding pyrrole[2] (2 equiv, 0.6 mmol). Over the resulting mixture was added HCl (MeOH) (2 equiv, 0.6 mmol) dropwise during 15 minutes. The obtained solution was stirred until aluminium oxide TLC (Hexane:AcOEt 3:1) analysis indicated complete consumption of the starting material. The solvent was evaporated at reduced pressure. The remaining residue was purified by column chromatography on aluminium oxide (hexane/AcOEt 3:1) to afford the corresponding triazole-prodiginine synthetic analogs **1a**.HCl–**1d**.HCl (Scheme S2).

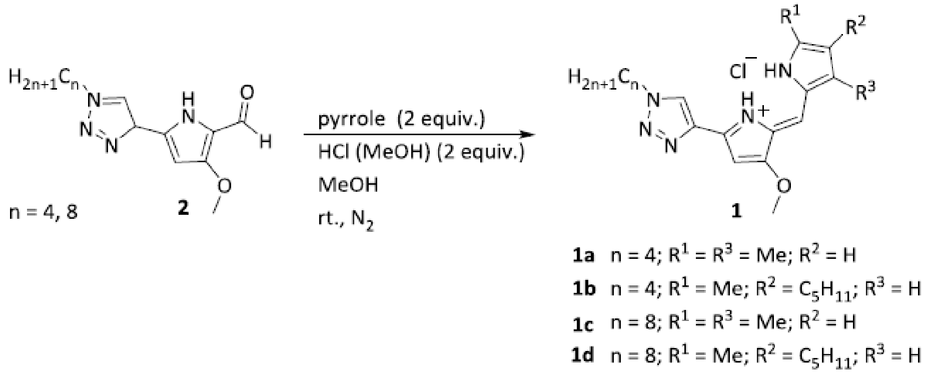

Supplementary Figure 2. Synthesis of triazole-prodiginine analogs **1a**.HCl–**1d**.HCl

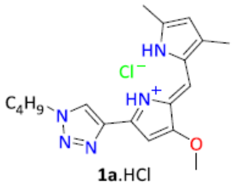

1a.HCl

5-(1-butyl-1*H*-1,2,3-triazol-4-yl)-2-((3,5-dimethyl-1*H*-pyrrol-2-yl)methylene)-3-methoxy-2*H*-pyrrol-1-ium chloride. **1a**.HCl. Yield 92 %. ^{1}H NMR (300 MHz, $CDCl_3$): δ = 13.59 (br s, NH, 1H), 13.29 (br s, NH, 1H), 9.72 (s, 1H), 7.27 (s, 1H), 6.69 (d, *J* = 1.2 Hz, 1H), 6.14 (s, 1H), 4.45 (t, *J* = 7.3 Hz, 2H), 4.05 (s, 3H, OCH_3), 2.62 (s,

3H), 2.34 (s, 3H), 2.02–1.92 (m, 2H), 1.46–1.34 (m, 2H), 0.97 (t, *J* = 7.3 Hz, 3H). ^{13}C NMR (75 MHz, $CDCl_3$): δ = 165.61 (C), 153.52 (C), 145.58 (C), 144.73 (C), 138.54 (C), 126.60 (CH), 126.43 (C), 119.16 (C), 117.19 (CH), 116.91 (CH), 94.58 (CH), 58.99 (OCH_3), 50.60 (CH_2), 32.17 (CH_2), 19.77 (CH_2), 14.41 (CH_3), 13.54 (CH_3), 12.17 (CH_3). HRMS (EI) m/z $[M]^+$ calc for [$C_{18}H_{23}N_5O$] 325.1903; found: 325.1913.

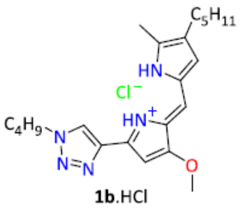

1b.HCl

5-(1-butyl-1*H*-1,2,3-triazol-4-yl)-3-methoxy-2-((5-methyl-4-pentyl-1*H*-pyrrol-2-yl)methylene)-2*H*-pyrrol-1-ium chloride. 1b.HCl. Yield: 91 %. ^{1}H NMR (300 MHz, $CDCl_3$): δ = 13.77 (br s, NH, 1H), 13.29 (br s, NH, 1H), 9.70 (s, 1H), 7.18 (s, 1H), 6.82 (d, *J* = 2.0 Hz, 1H), 6.68 (d, *J* = 1.8 Hz, 1H), 4.45 (t, *J* = 7.3 Hz, 2H), 4.03 (s, 3H, OCH_3), 2.60 (s, 3H), 2.40 (t, *J* = 7.6 Hz, 2H), 2.02–1.92 (m, 2H), 1.60–1.50 (m, 2H), 1.44–1.30 (m, 6H), 0.97 (t, *J* = 7.4 Hz, 3H), 0.89 (t, *J* = 6.9 Hz, 3H). ^{13}C NMR (75 MHz, $CDCl_3$): δ = 165.76 (C), 152.78 (C), 145.75 (C), 138.43 (C), 131.51 (CH), 131.07 (C), 126.61 (CH), 126.46 (C), 119.91 (CH), 119.68 (C), 94.57 (CH), 58.99 (OCH_3), 50.56 (CH_2), 32.11 (CH_2), 31.43 (CH_2), 29.48 (CH_2), 25.36 (CH_2), 22.51 (CH_2), 19.72 (CH_2), 14.07 (CH_3), 13.49 (CH_3), 12.81 (CH_3). HRMS (EI) m/z $[M]^+$ calcd for [$C_{22}H_{31}N_5O$] 381.2529; found: 381.2524.

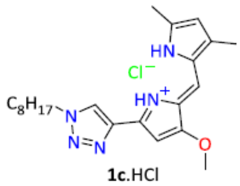

1c.HCl

2-((3,5-dimethyl-1*H*-pyrrol-2-yl)methylene)-3-methoxy-5-(1-octyl-1*H*-1,2,3-triazol-4-yl)-2*H*-pyrrol-1-ium chloride. 1c.HCl. Yield: 89 %. ^{1}H NMR (300 MHz, $CDCl_3$): δ = 13.54 (br s, NH, 1H), 13.24 (br s, NH, 1H), 9.67 (s, 1H), 7.21 (s, 1H), 6.64 (d,*J* = 1.9 Hz, 1H), 6.09 (s, 1H), 4.41 (t, *J* = 7.3 Hz, 2H), 4.00 (s, 3H, OCH_3), 2.58 (s, 3H), 2.30 (s, 3H), 1.99–1.90 (m, 2H), 1.31–1.30 (m, 4H), 1.25–1.22 (m, 6H), 0.86 (t, *J* = 6.7 Hz, 3H). ^{13}C NMR (75 MHz, $CDCl_3$): δ = 165.65 (C), 153.55 (C), 145.67 (C), 144.74 (C), 138.59 (C), 126.62 (CH), 126.48 (C), 119.23 (C), 117.24 (CH), 116.94 (CH), 94.64 (CH), 59.02 (OCH_3), 50.97 (CH_2), 31.84 (CH_2), 30.34 (CH_2), 29.18 (CH_2), 29.08 (CH_2), 26.58 (CH_2), 22.73 (CH_2), 14.46 (CH_3), 14.21 (CH_3), 12.20 (CH_3). HRMS (EI) m/z $[M]^+$ calcd for [$C_{22}H_{31}N_5O$] 381.2529; found: 381.2524.

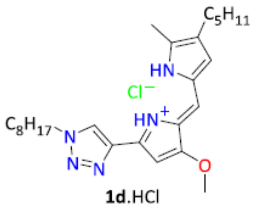

1d.HCl

3-methoxy-2-((5-methyl-4-pentyl-1*H*-pyrrol-2-yl)methylene)-5-(1-octyl-1*H*-1,2,3-triazol-4-yl)-2*H*-pyrrol-1-ium chloride. 1d.HCl. Yield: 94 %. ^{1}H NMR (300 MHz, $CDCl_3$): δ = 13.76 (br s, NH, 1H), 13.29 (br s, NH, 1H), 9.68 (s, 1H), 7.17(s, 1H), 6.82 (d, *J* = 2.1 Hz, 1H), 6.67 (d, *J* = 1.9 Hz, 1H), 4.43 (t, *J* = 7.3 Hz, 2H), 4.02 (s, 3H, OCH_3), 2.59 (s, 3H), 2.39 (t, *J* = 7.5 Hz, 2H), 2.02–1.92 (m, 2H), 1.59–1.49 (m, 2H), 1.33–1.24 (m, 14H), 0.89 (t, *J* = 6.9 Hz, 3H), 0.86 (t, *J* = 6.9 Hz, 3H). ^{13}C NMR (75 MHz, $CDCl_3$): δ = 165.84 (C), 152.86 (C), 145.86 (C), 138.52 (C), 131.57 (CH), 131.15 (C), 126.65 (CH), 126.53 (C), 120.00 (CH), 119.77 (C), 94.66 (CH), 59.05 (OCH_3), 50.96 (CH_2), 31.82 (CH_2), 31.50 (CH_2), 30.31 (CH_2), 29.57 (CH_2), 29.17 (CH_2), 29.06 (CH_2), 26.57 (CH_2), 25.44 (CH_2), 22.71 (CH_2), 22.59 (CH_2), 14.19 (CH_3), 14.15 (CH_3), 12.90 (CH_3). HRMS (EI) m/z $[M]^+$ calc for $[C_{26}H_{39}N_5O]$ 437.3155; found: 437.3159.

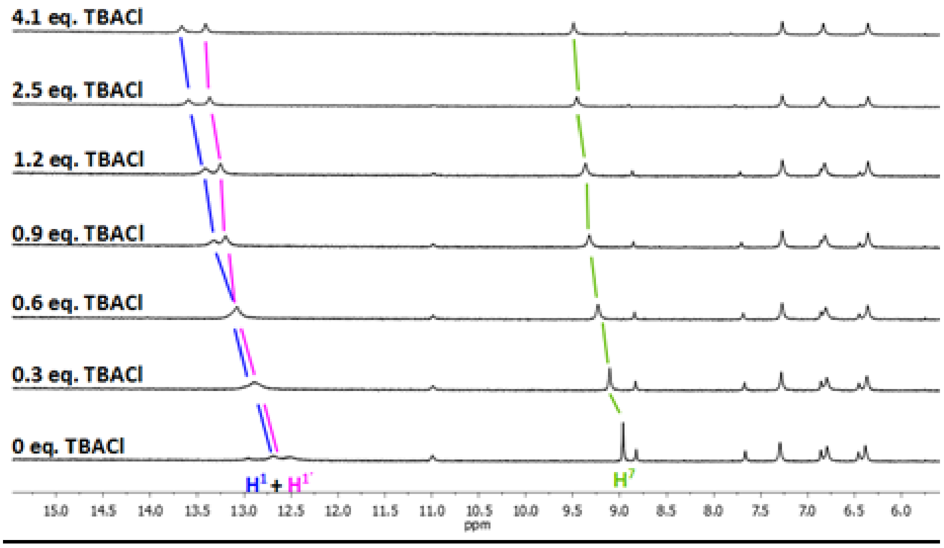

Supplementary Figure 3. Partial stack plot of ^{1}H NMR spectra of compound **1a**.$HClO_4$ in DMSO-d_6-0.5% H_2O solution under the addition of increasing amounts of tetrabutylammonium chloride.

X-ray structure 1A.HCL. A single crystal of **1a**.HCl was coated in high-vacuum grease and mounted on a glass fibre. X-ray measurements were made using a Bruker SMART CCD area-detector diffractometer with Mo-K_{α} radiation (λ = 0.71073 Å). Intensities were integrated from several series of exposures, each exposure covering 0.3° in ω, and the total data set being a hemisphere. Absorption corrections were not applied. The structure was solved by direct methods and refined by least squares on weighted F^2 values for all reflections (Table S3).

All non-hydrogen atoms were assigned anisotropic displacement parameters and refined without positional constraints. Hydrogen atoms (**H1, H2)** were located in the electron density difference map, assigned isotropic displacement parameters and refined without positional constraints. All other hydrogen atoms were constrained to ideal geometries and refined with fixed isotropic displacement parameters. Refinement proceeded smoothly to give the residuals shown in Table S1. Complex neutral-atom scattering factors were used[3].

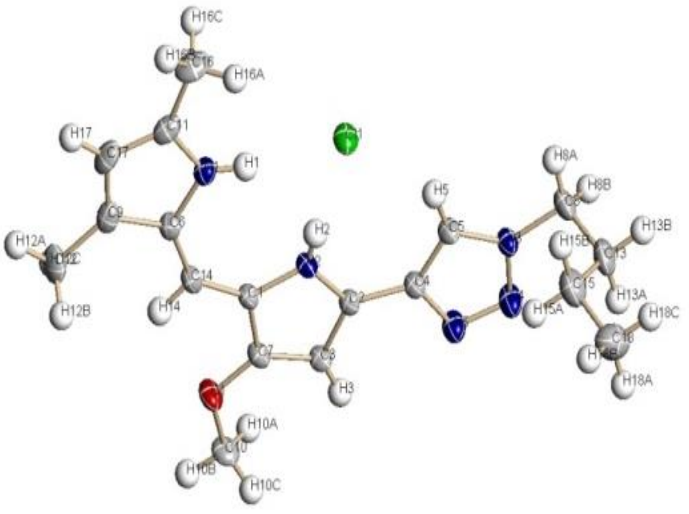

Supplementary Figure 4. X-ray structure of compound **1a**.HCl

Supplementary Table 1. Crystal data and structure refinement for **1a**.HCl

Identification code	datosm	
Empirical formula	C_{18} H_{24} Cl N_5 O	
Formula weight	361.87	
Temperature	173(2) K	
Wavelength	0.71073 Å	
Crystal system	Monoclinic	
Space group	C2/c	
Unit cell dimensions	a = 24.912(10) Å	α = 90°
	b = 7.390(3) Å	β =109.972(7)°
	c = 21.639(9) Å	γ = 90°
Volume	3744(3) $Å^3$	
Z	8	
Density (calculated)	1.284 Mg/m^3	
Absorption coefficient	0.220 mm^{-1}	
F(000)	1536	
Crystal size	0.6 x 0.2 x 0.2 mm	
□ range for data collection	1.74 to 25.00°	
Index ranges	$-29 \le h \le 29$, $-8 \le k \le 8$, $-25 \le l \le 25$	
Reflections collected	17645	
Independent reflections	3299 [R_{int} = 0.1668]	
Completeness to θ = 25.00°	100.0 %	
Refinement method	Full-matrix least-squares on F^2	
Data / restraints / parameters	3299 / 0 / 238	
Goodness-of-fit on F^2	S = 1.025	
R indices [for 2654 reflections with I>2σ(I)]	R_1 = 0.0539, wR_2 = 0.1319	
R indices (for all 3299 data)	R_1 = 0.0644, wR_2 = 0.1373	
Weighting scheme	$w^{-1} = \sigma^2(F_o^2) + (aP)^2 + (bP)$,	
	where $P = [\max(F_o^2, 0) + 2F_c^2]/3$	
	a = 0.0631, b = 0.000	
Largest diff. peak and hole	0.512 and -0.335 $eÅ^{-3}$	

<u>Computational methods</u>. Calculations of the molecular electronic structure were done using the Gaussian 09 program[4]. The geometries of all species were fully optimized at the B3LYP/6-31+G** level. The environmental effects were taken into account by the Polarizable Continuum Mode (PCM) using the CPCM model[5]. The nature of all optimized structures was determined using harmonic frequency analysis as true minima with no imaginary frequencies.

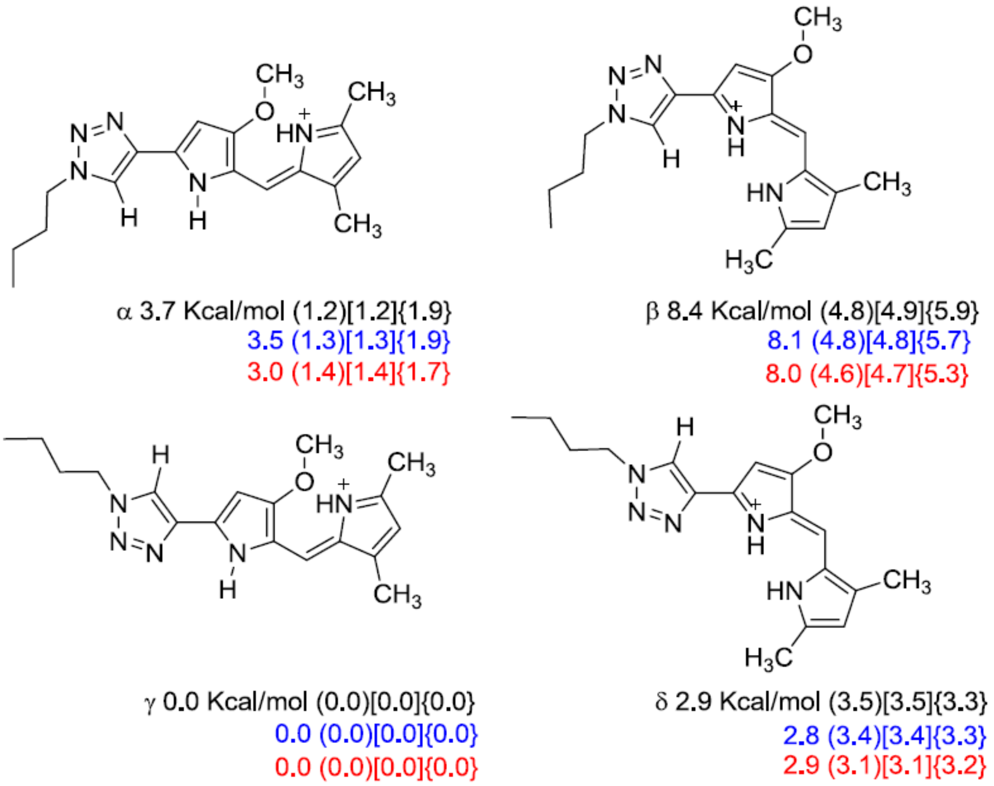

Supplementary Figure 5. Relative energies for the rotamers of protonated prodiginine **1a.**H^+. *Total energy, enthalpy and Gibbs free energy*, in kcal/mol, are calculated in gas phase, water (parentheses), DMSO [square brackets] and chloroform {curly brackets}

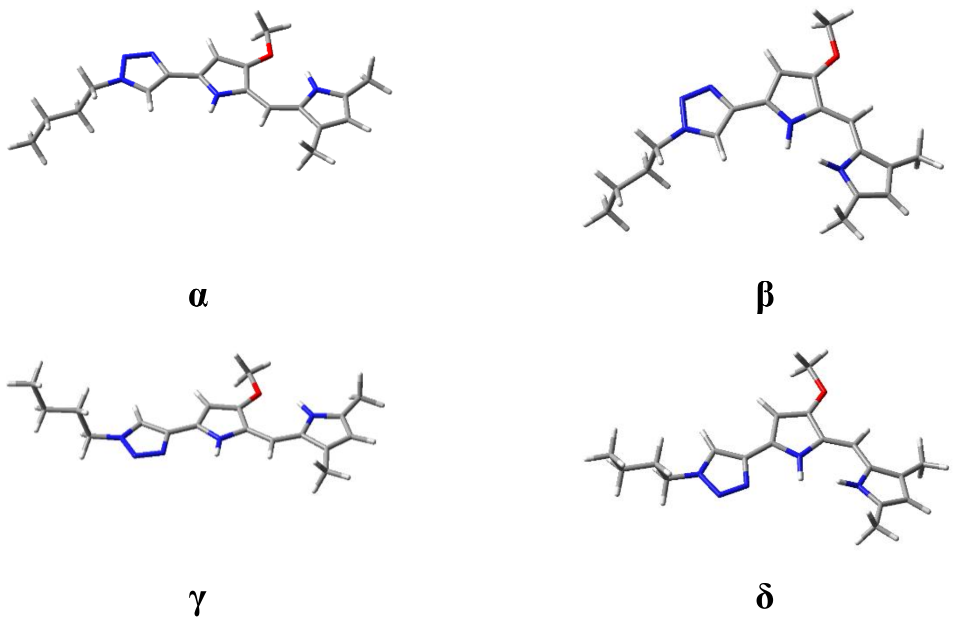

Supplementary Figure 6. Optimized geometries (gas phase) for rotamers of **1a**H$^+$

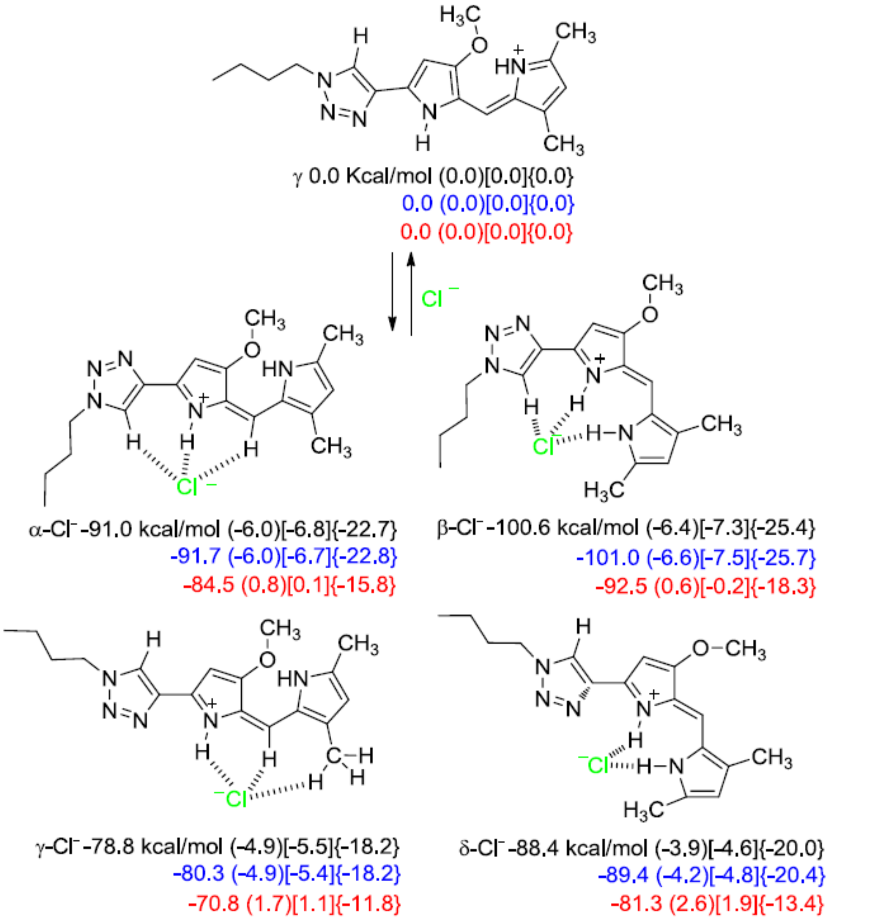

Supplementary Figure 7. Calculated binding energies for **1a.**H$^+$ towards chloride anion. *Total energy, enthalpy and Gibbs free energy*, in kcal/mol, are calculated in gas phase, water (parentheses), DMSO [square brackets] and chloroform {curly brackets}.

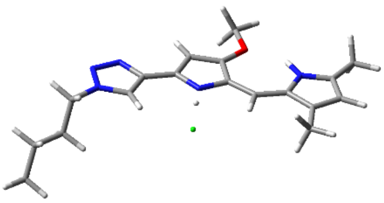

α-Cl⁻

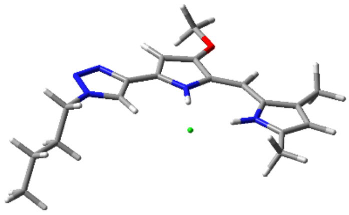

β-Cl⁻

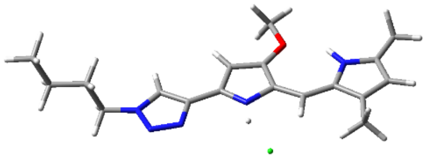

γ-Cl⁻

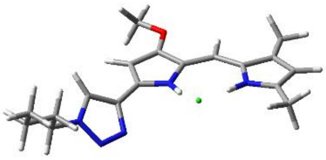

δ-Cl⁻

Supplementary Figure 8. Optimized geometries (gas phase) for supramolecular complexes of **1a.**$H^+$with chloride anion.

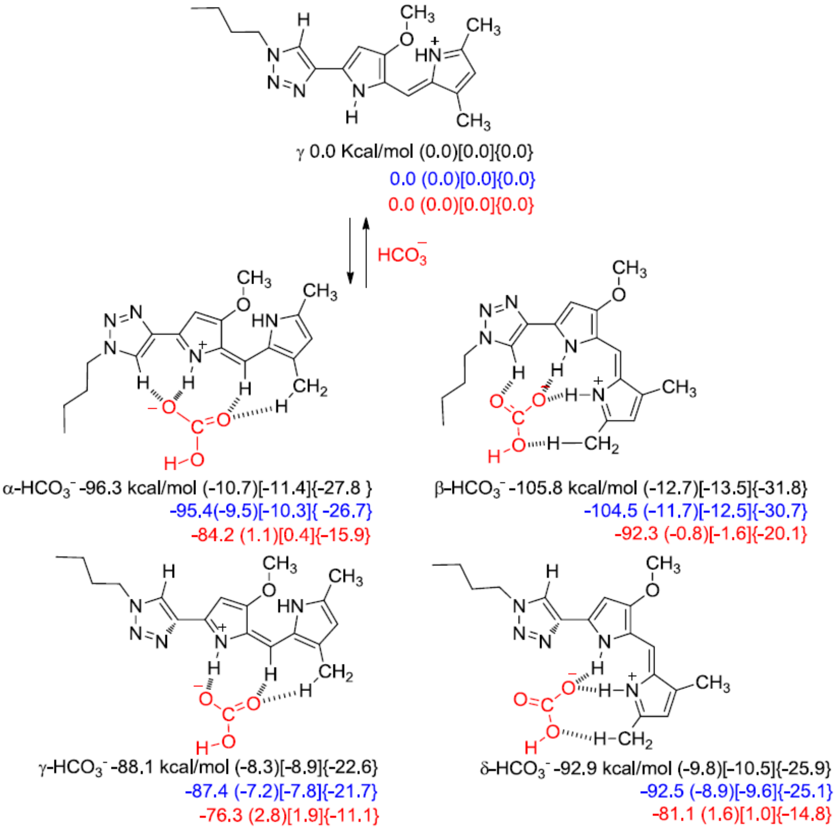

Supplementary Figure 9. Calculated binding energies for **1a.**H^+ towards hydrogencarbonate anion. *Total energy, enthalpy and Gibbs free energy*, in kcal/mol, are calculated in gas phase, water (parentheses), DMSO [square brackets] and chloroform {curly brackets}.

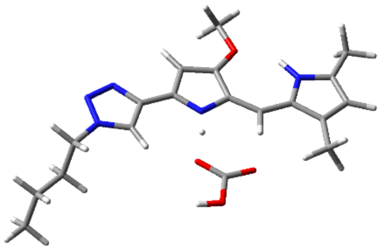

α-HCO_3^-

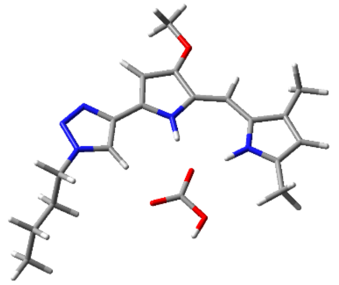

β-HCO_3^-

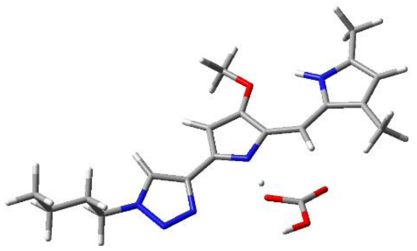

γ-HCO_3^-

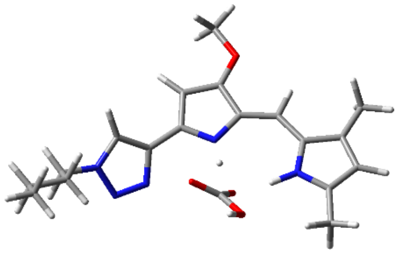

δ-HCO_3^-

Supplementary Figure 10. Optimized geometries (gas phase) for supramolecular complexes of **1a.**H$^+$with hydrogencarbonate anion.

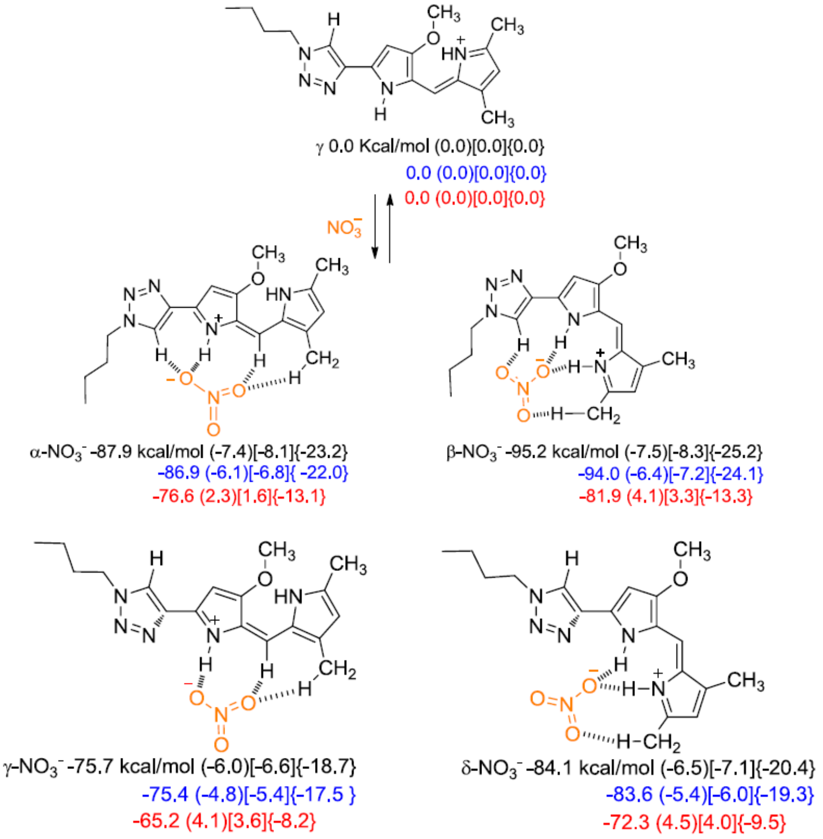

Supplementary Figure 11. Calculated binding energies for **1a.**H$^+$ towards nitrate anion. *Total energy, enthalpy and Gibbs free energy*, in kcal/mol, are calculated in gas phase, water (parentheses), DMSO [square brackets] and chloroform {curly brackets}.

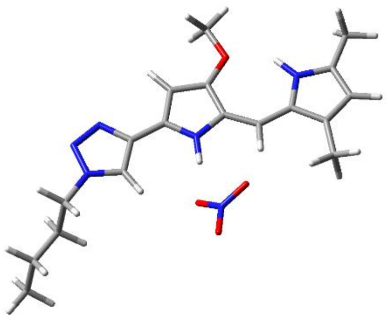

α-NO_3^-

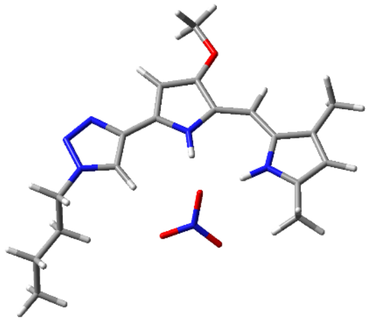

β-NO_3^-

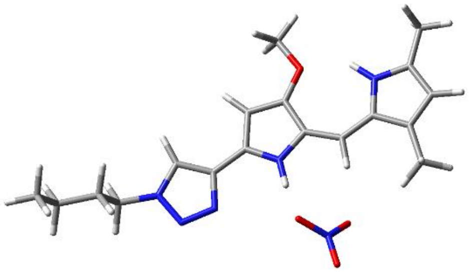

γ-NO_3^-

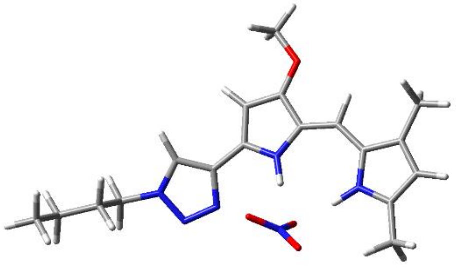

δ-NO_3^-

Supplementary Figure 12. Optimized geometries (gas phase) for supramolecular complexes of **1a.**$H^+$with nitrate anion.

Anion transport studies: chloride Selective Electrode (ISE) assays

Preparation of large unilamelar vesicles (LUV). A chloroform solution (20 mg/mL) of 1-palmitoyl-2-oleoyl-sn-glycero-3- phosphocholine (POPC) (Sigma-Aldrich) was evaporated *in vacuo* in a rotary evaporator and the lipid film obtained was dried under high vacuum for at least 4 hours. The lipid film was rehydrated by careful vortexing with a solution containing 489 mM NaCl, ionic strength 500 mM and 5 mM phosphate buffer, pH 7.2 or 451 mM NaCl, ionic strength 500 mM and 20 mM phosphate buffer, pH 7.20. The lipid suspension was subjected to nine freeze-thaw cycles, freezing it in a liquid nitrogen bath and thawing it in a mild water bath alternatively. Then, the phospholipid suspension was extruded 29 times through a 200 nm polycarbonate membrane[6] using a LiposoFast Basic extruder (Avestin, Inc.). Finally, the obtained LUV obtained were dialyzed against a nitrate (489 mM $NaNO_3$, ionic strength 500 mM and 5 mM phosphate buffer, pH 7.2) or sulfate (150 mM Na_2SO_4 mM, ionic strength 500 mM and 20 mM phosphate buffer, pH 7.2), solution, to remove the unencapsulated chloride. Finally, the LUV were brought to a known volume with the nitrate or sulfate solution, respectively.

For the valinomycin experiments, 20 mg/ml of Asolectin from soybean (Sigma-Aldrich 11145, mixture of phospholipids) were dissolved in chloroform, and lipid films were obtained by evaporation of the solvent under a gentle nitrogen flux; in order to remove all chloroform, films were further dried overnight in vacuum. The phospholipids were hydrated in chloride buffer (in mM: NaCl 450, 20 mM HEPES; pH 7.0), and vigorously vortex mixed and, to ensure equilibration, sonicated in 5 cycles of 1.5 min each, with 1 min rest, in ice. Liposomes were centrifuged at 2000 g for 5–10 minutes to remove any titanium particle released by the sonicator tip and larger aggregates. LUV were then obtained by extrusion through polycarbonate filters mounted in extruder. Samples were subjected to 19 passes through a single 100 nm mesh filter. External solution was exchanged twice in a Sephadex G25 column previously equilibrated with the external chloride-free solution in mM: $NaNO_3$ 450, 20 mM HEPES; pH 7.0).

Chloride efflux measurements in LUV. Efflux of chloride from LUV was measured with a chloride ion-sensitive electrode (Vernier, Beaverton, Oregon, USA) in a constantly stirred 3.5 to 5.0 ml LUV suspension. Data was acquired using a LabQuest mini interface (Vernier). Ionophores were dissolved in DMSO to a concentration of 10 mM. After an initial equilibration, chloride efflux was induced by a small volume (<1%) of ionophore. Control experiments where similar amounts of DMSO (without anionophores) were added demonstrated that these concentrations of DMSO do not induce any chloride efflux (see below). The measurement was concluded with the addition of the detergent Triton-X or polyoxyethylene 10 tridecyl ether (C13E10) to break off the bilayers and measure the maximum chloride content in the LUV's.

Determination of the dose-response (EC_{50}). The normalised chloride efflux at 300 s against carrier concentration were fitted with the Hill equation:

$$\frac{Cl(300)}{Cl_{max}} = \frac{A^n}{\mathrm{EC}_{50}^n + A^n}$$

where $Cl(300) / Cl_{max}$ is the ratio between the external chloride concentration after 300 s and the maximum chloride concentration upon application of detergent; A is the concentration of the anion carrier; EC_{50} is the concentration to obtain the half of the maximum effect; n is the Hill's coefficient.

Figures S10-S13 show the transport data driven by the application of **1a-d** and prodigiosine on LUV containing NaCl (489 mM NaCl and 5 mM phosphate buffer, pH 7.2), and immersed in $NaNO_3$ (489 mM $NaNO_3$ and 5 mM phosphate buffer, pH 7.2). At the end of the experiment the vesicles were lysed with detergent to release all chloride ions and the resulting value was considered to represent the 100% and was used as such. Each trace represents the average of at least three different experiments, carried out with at least three different batches of vesicles.

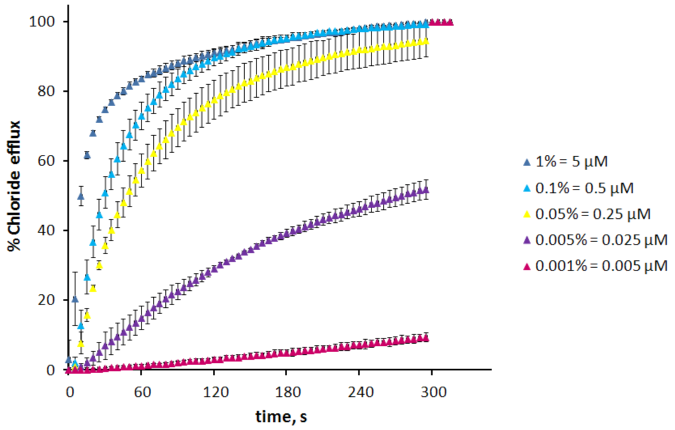

Supplementary Figure 13. Chloride efflux upon addition of **1a**.HCl (5 µM, 1 mol%; 0.5 µM, 0.1 mol%; 0.25 µM, 0.05 mol%; 0.025 µM, 0.005 mol% and 0.005 µM, 0.001% carrier to lipid)

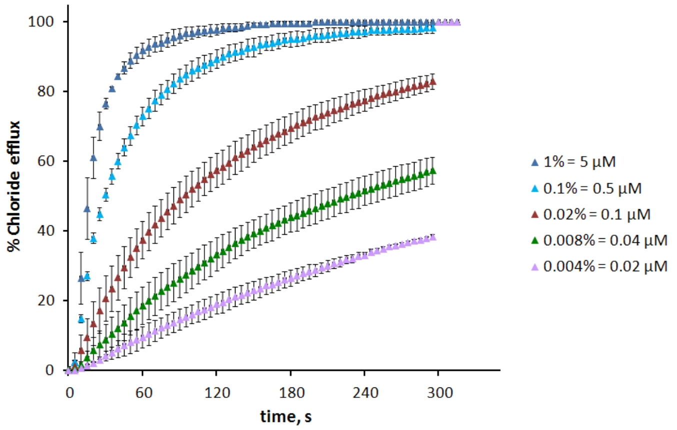

Supplementary Figure 14. Chloride efflux upon addition of **1b**.HCl (5 µM, 1 mol%; 0.5 µM, 0.1 mol%; 0.25 µM, 0.05 mol%; 0.025 µM, 0.005 mol% and 0.005 µM, 0.001% carrier to lipid)

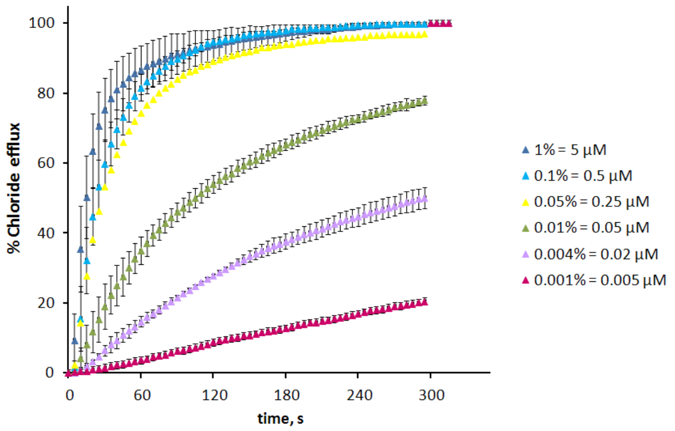

Supplementary Figure 15. Chloride efflux upon addition of **1c**.HCl (5 µM, 1 mol%; 0.5 µM, 0.1 mol%; 0.25 µM, 0.05 mol%; 0.025 µM, 0.005 mol% and 0.005 µM, 0.001% carrier to lipid)

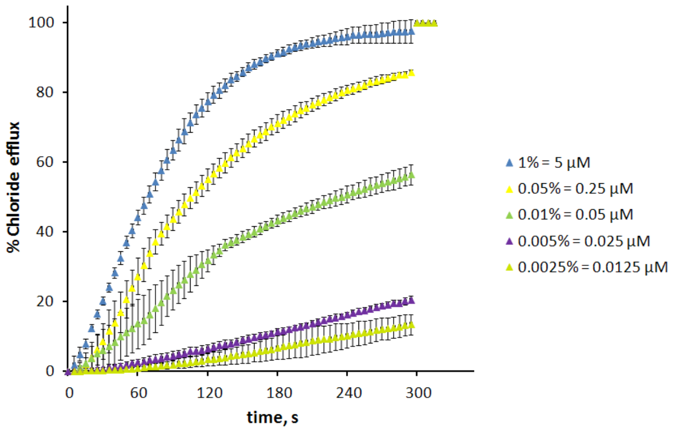

Supplementary Figure 16. Chloride efflux upon addition of **1d**.HCl (5 µM, 1 mol%; 0.5 µM, 0.1 mol%; 0.25 µM, 0.05 mol%; 0.025 µM, 0.005 mol% and 0.005 µM, 0.001% carrier to lipid)

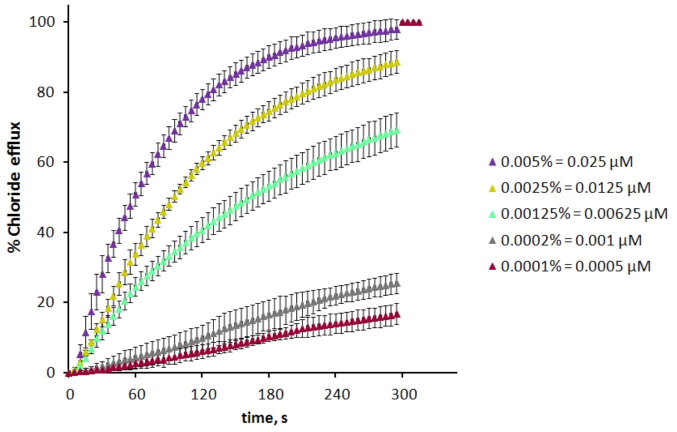

Supplementary Figure 17. Chloride efflux upon addition of prodigiosine (5 µM, 1 mol%; 0.5 µM, 0.1 mol%; 0.25 µM, 0.05 mol%; 0.025 µM, 0.005 mol% and 0.005 µM, 0.001% carrier to lipid)

Figures S15-S19 show the transport data driven by the application of **1a-d** and prodigiosine on LUV containing 451 mM NaCl and 20 mM phosphate buffer, pH 7.2, were immersed in150 mM Na_2SO_4, 40 mM HCO_3^- and 20 mM phosphate buffer, pH 7.2. At the end of the experiment the vesicles were lysed with detergent to release all chloride ions and the resulting value was considered to represent the 100% and was used as such. Each trace represents the average of at least three different experiments, carried out with at least three different batches of vesicles.

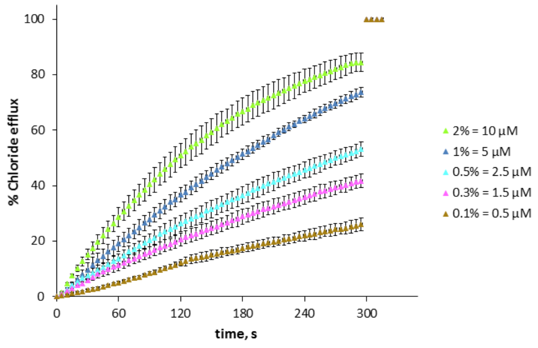

Supplementary Figure 18. Chloride efflux upon addition of prodigiosine **1a.**HCl (10 µM, 2 mol%; 5 µM, 1 mol%; 2.5 µM, 0.5 mol%; 1.5 µM, 0.3 mol% and 0.5 µM, 0.1 mol% carrier to lipid)

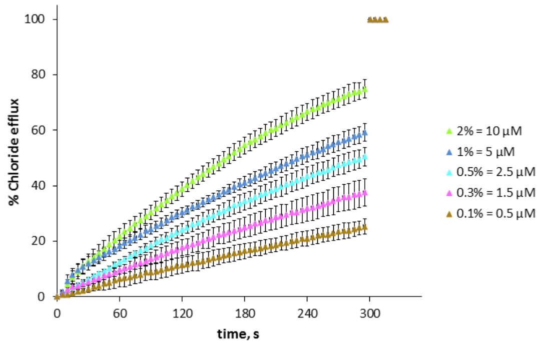

Supplementary Figure 19. Chloride efflux upon addition of prodigiosine **1b.**HCl (10 µM, 2 mol%; 5 µM, 1 mol%; 2.5 µM, 0.5 mol%; 1.5 µM, 0.3 mol% and 0.5 µM, 0.1 mol% carrier to lipid)

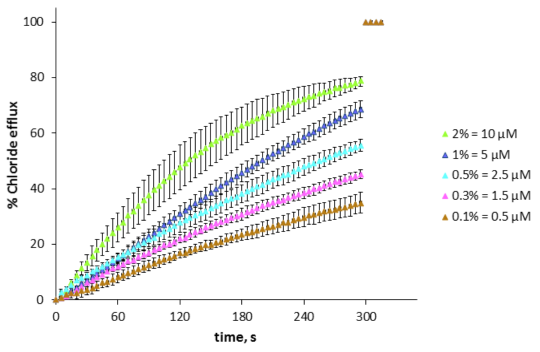

Supplementary Figure 20. Chloride efflux upon addition of prodigiosine **1c.**HCl (10 µM, 2 mol%; 5 µM, 1 mol%; 2.5 µM, 0.5 mol%; 1.5 µM, 0.3 mol% and 0.5 µM, 0.1 mol% carrier to lipid)

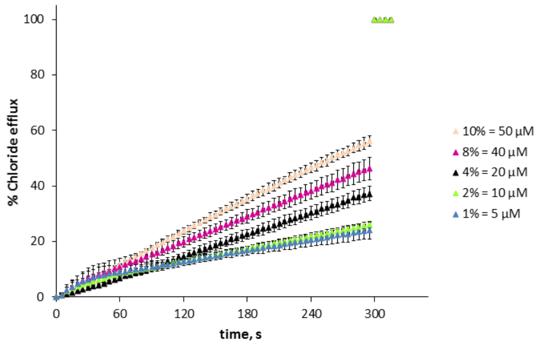

Supplementary Figure 21. Chloride efflux upon addition of prodigiosine **1d.**HCl (10 µM, 2 mol%; 5 µM, 1 mol%; 2.5 µM, 0.5 mol%; 1.5 µM, 0.3 mol% and 0.5 µM, 0.1 mol% carrier to lipid)

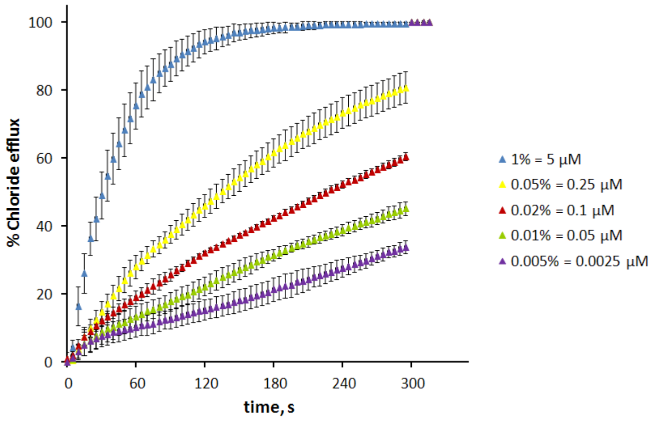

Supplementary Figure 22. Chloride efflux upon addition of prodigiosine **prodigiosine**(10 µM, 2 mol%; 5 µM, 1 mol%; 2.5 µM, 0.5 mol%; 1.5 µM, 0.3 mol% and 0.5 µM, 0.1 mol% carrier to lipid)

Anion transport studies: Lucigenin based fluorescence assays. LUV composed of mixture of POPC and cholesterol, at a ratio of 7:3. Phospholipid were rehydrated with 102.2 mM $NaNO_3$, 20 mM phosphate buffer and Lucigenin 3 mM, pH 7.2) The non-encapsulated lucigenin was removed by size exclusion chromatography, using a Sephadex G-50 column, eluted with a lucigenin-free nitrate solution. The fluorescence emission at 503 nm after excitation at 372 nm was recorded using a Hitachi F-7000 fluorescence spectrophotometer, at 25 ^{o}c. After recording the fluorescence baseline for 60 s, an aliquot of the anion carrier in methanol was added. The fluorescence emission was then recorded during five minutes.

Safranin O assays. LUV were prepared as described above. Phospholipids were rehydrated with a chloride solution, and the non-encapsulated solution was replaced by sulphate by size exclusion chromatography. At the beginning of the assay Safranin O was added for a final concentration of 0.2 µM.The fluorescence at 580 nm after exciting at 520 nm was recorded for two minutes, and an aliquote of theanion carrier, in DMSO solution, was added; fluorescence changes were monitored over time for 10 minutes. The fluorescence intensity relationship I(t) / I(120) was plotted as a function of time.

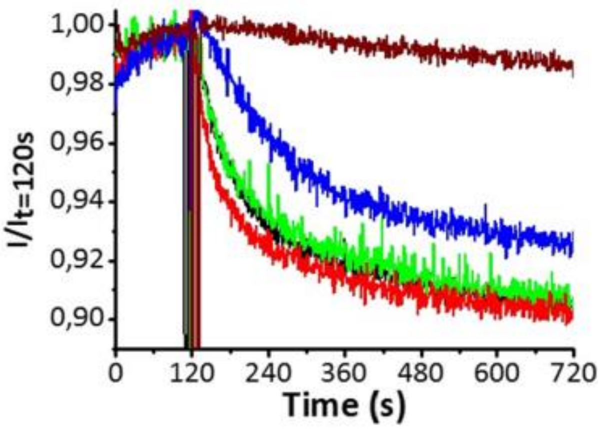

Supplementary Figure 23. Safranin O assay upon addition of **1a**-**d** to POPC vesicles, 0.25 mM. Vesicles contained NaCl (28.8 mM) were suspended in Na2SO4 (9.6 mM) solution with Safranin O 0.2 µM. At t=120 s the compounds were added to the external solution. Black trace: **1a**.HCl, green trace: **1b**.HCl, red trace: **1c**.HCl, blue trace: **1d**.HCl and brown trace: blank (10 µL DMSO).

Cell lines and culture conditions

Human lung adenocarcinoma (A549) cells were cultured in DMEM medium (Biological Industries, Beit Haemek, Israel) supplemented with 10% heat-inactivated foetal bovine serum (FBS; Life Technologies, Carlsbad, CA), 100 U/mL penicillin, 100 µg/mL streptomycin, and 2 mM L-glutamine, all from Biological Industries. Cells were grown at 37 °C under a 5% CO_2 atmosphere.

Human mammary adenocarcinoma (MCF-7) cell line was cultured in DMEM–F12 media (1:1, Biological Industries) supplemented with 10% heat-inactivated FBS, 100 nM sodium pyruvate (Biological Industries), 10 µg/mL insulin (Sigma-Aldrich, St. Louis, MO), 100 U/mL penicillin, 100 µg/mL streptomycin, and 2 mM L-glutamine. Cells were grown at 37 °C under a 5% CO_2 atmosphere.

Human mammary epithelial (MCF-10A) cell line was cultured in DMEM–F12 media (1:1) supplemented with 5% horse serum (Life Technologies), 20 ng/mL EGF, 0.5 µg/mL hydrocortisone, 100 ng/mL cholera toxin, 10 µg/mL insulin, all from Sigma-Aldrich, and 100 U/mL penicillin, 100 µg/mL streptomycin, and 2 mM L-glutamine. Cells were grown at 37 °C under a 5% CO_2 atmosphere.

Fisher Rat Thyroid (FRT) cells stably transfected with a halide-sensitive yellow fluorescence protein (YFP-H148Q/I152L)[7] cells were cultured on black-wall, clear bottom 96-well micro-plates at a density of 40,000 cells/well in Coon's modified medium supplemented with 10% serum, 2 mM L-glutamine, 1 mg/ml penicillin, 100 µg/ml streptomycin, and 0.5 mg/ml hygromycin as selection agent for the YFP. Cells were maintained at 37° C in a 5% CO_2/95% air atmosphere. For some experiments, cells co-transfected with the YFP and with a wild type CFTR were used. In this case the culture medium contained 1 mg/ml G418 and 0.6 mg/ml zeocin as selection agents. Functional experiments were done 48 h after cell seeding.

Human embryonic kidney (HEK) cell lines were grown in standard conditions, in Ham's F10 medium supplemented with 2 mM L-glutamine and 10% foetal bovine serum. All culture reagents were purchased from Sigma Aldrich. Cells were grown at 37 °C under a 5% CO_2 atmosphere.

Cell viability assays. Cell viability was determined by the MTT assay. Briefly, cells (1×10^5 cells/mL) were seeded in 96-well plates and allowed to grow for 24 h. Afterwards, dose–response experiments were performed treating cells with 5, 10 and 20 µM of each compound for 24 h. Prodigiosin was used as a cytotoxic positive control compound. DMSO (diluent) was added in control cells. After treatment, 10 µM of 3-(4,5-dimethylthiazol-2-yl)-2,5-diphenyltetrazolium bromide (Sigma-Aldrich) was added to each well for an additional 4 h. Media was removed and the blue MTT formazan precipitate was dissolved in 100 µl of DMSO. The absorbance at 570 nm was measured on a Multiskan FC multiwell plate reader (Thermo Scientific Inc., Waltham, MA). Cell viability was expressed as a percentage of control cells. Data are shown as the mean value ± standard deviation of four independent experiments performed in duplicate.

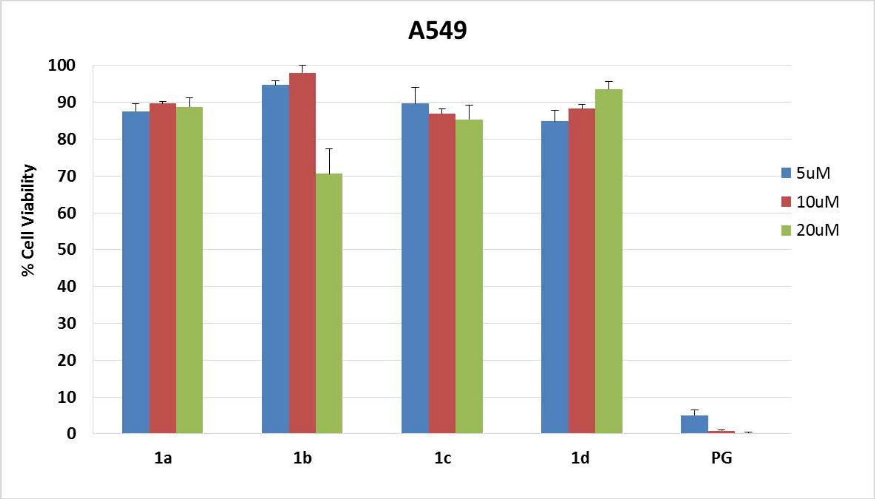

Supplementary Figure 24.. A549 cell viability after treatment with **1a** -**1d**.

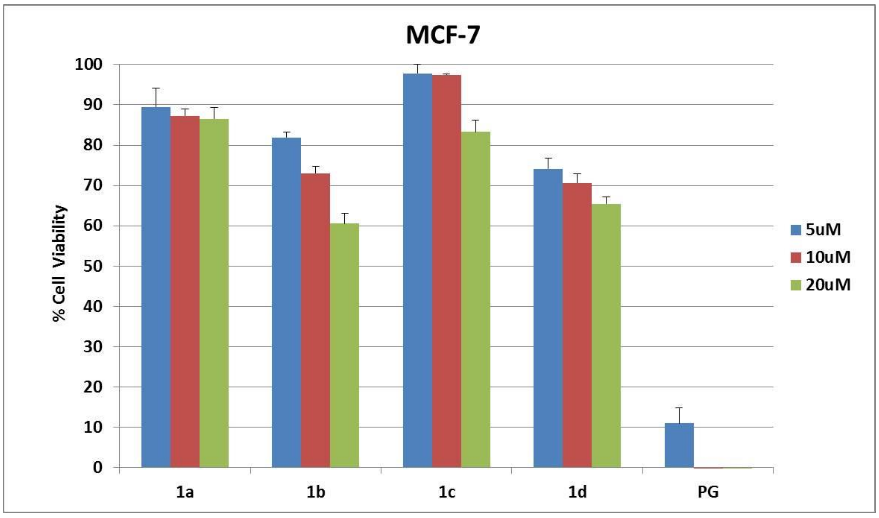

Supplementary Figure 25. MCF-7 cell viability after treatment with **1a -1d**.

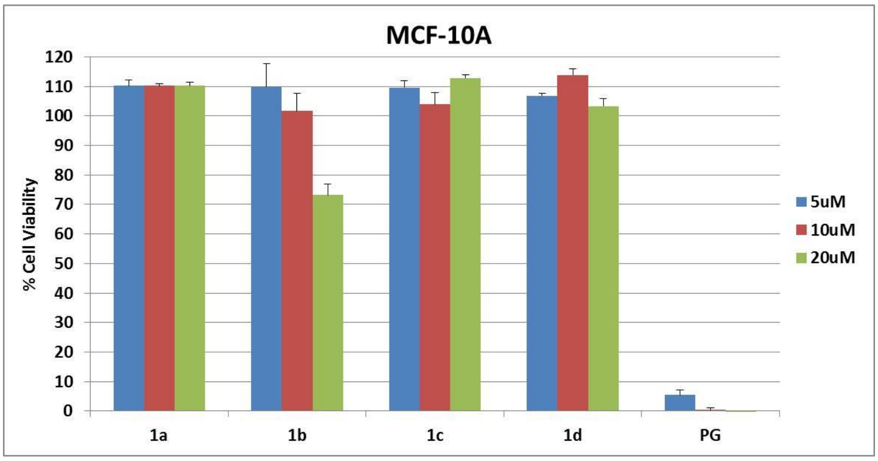

Supplementary Figure 26.. MCF-10A cell viability after treatment with **1a -1d**.

Intracellular pH measurement. Measurement of intracellular pH was done by flow cytometry using a ratiometric fluorophore. A549 cells (10^6 cells/mL) were incubated with 20 µM of SNARF-1-AM (Molecular Probes, Thermo Fisher Scientific Inc., MA) in PBS for 1 h at 37 ºC with intermittent shaking. Then, 5×10^5 cells were resuspended in high-potassium buffered at various pH values, from 6.2 to 8.0 containing 2 µg/mL of nigericin (Sigma-Aldrich) to obtain a calibration curve. For test, A549 cells were treated with 10 µM of selected compounds for 1 h. Then, control and treated cell suspensions were incubated with SNARF-1-AM in the same conditions as above, and resuspended in PBS. SNARF-1 fluorescence emission was collected at 576 and 664 nm using a MoFlo Astrios cell sorter cytometer (Beckman Coulter, Miami FL) and ratios (664/576) were calculated for the standard curve as well as for treated cells.

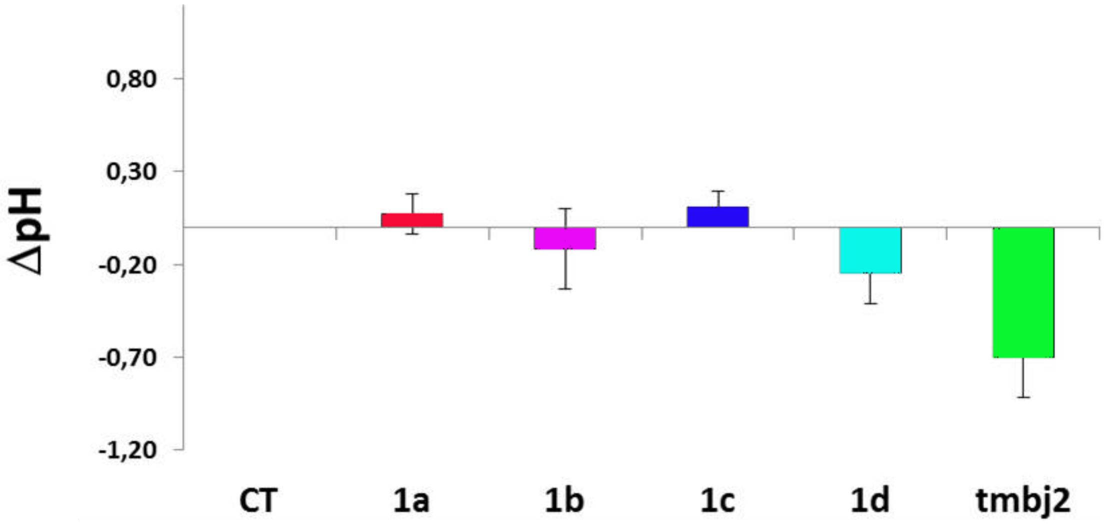

Supplementary Figure 27. Intracellular pH variations in A549 after being treated with **1a-1d.** Tambjamin, a natural porduct, was used as positive control.

Acridine Orange assay. A549 cells (10^5 cells/mL) were seeded onto glass coverslips in a 12-well plate and 24 h later they were treated with 10 µM of the studied compounds. DMSO (1% v/v) was added to control cells. After 1 h, the cells were washed twice with PBS and incubated in 5 µg/mL acridine orange solution (Sigma-Aldrich) for 30 min at room temperature. Finally, they were washed with PBS-10% FBS twice and fluorescence was examined with a NIKON eclipse E800 microscope (SCT filter 440/480 nm, Nikon Instruments, Melville, NY, USA).

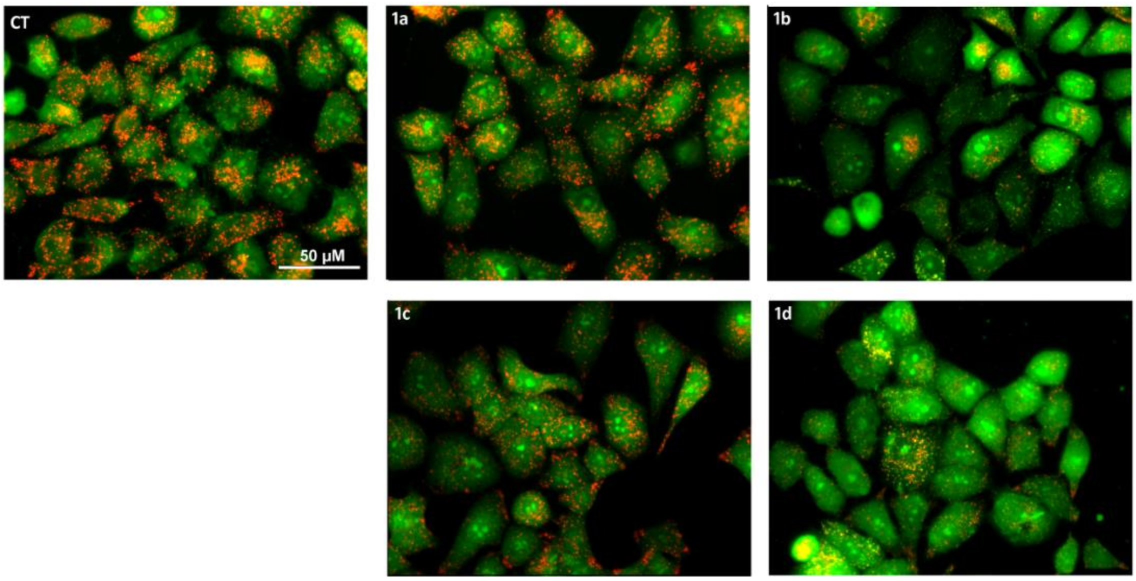

Supplementary Figure 28. Acridine orange assay in A549 after treatment with **1a** -**1d**.

Anion transport assays in living cells

Measurement of iodide influx in cells. FTR-cells expressing the halide-sensitive YFP protein were incubated in phosphate-buffered solution (PBS) containing (in mM): NaCl 137, KCl 2.7, Na_2HPO_4 8.1, KH_2PO_4 1.5, $CaCl_2$ 1 and $MgCl_2$ 0.5 (pH 7.3). The solution injected during the assay is similar but contained NaI 137 mM instead of NaCl (pH 7.3). To explore the effect of lowering the extracellular pH, the NaI solution was buffered at pH 6.9 with HEPES, or at pH 6.6 and 6.2 using MES. The activity of anionophores was determined using a fluorescence plate reader equipped with 500 nm excitation and 535 emission filters, as previously described[8]. The assay is based in the fact that the fluorescence of the YFP is greater quenched by iodide than by chloride7. Functional assays were done at 37°C.

If not otherwise stated, 30 minutes before the assay, the cells were washed twice with a PBS containing 137 mM NaCl. The cells were incubated in 60 µl PBS at 37°C with anionophores or with DMSO as control. Once the assay started, the fluorescence was

recorded every 0.2 seconds for as long as 14-40 s for each well. At 2 seconds after the start of fluorescence recording were injected 165 µl of a PBS containing 137 mM NaI instead of NaCl, so that the final concentration of NaI in the well is 100 mM. If the anionophore tested is active and allows the influx of I^-, this anion binds to the YFP and quenches the fluorescence. After background subtraction and normalization for the maximal value before NaI addition, the signal decay was fitted with a double exponential function and the maximum rate of fluorescence decay (QR) was derived. This parameter is a direct indication of the activity of the tested compound.

Measurement of chloride efflux in cells. HEK-293 cells were detached from the flask bottom by soft scrapping, washed in chloride-free solution, and used immediately. For chloride efflux measurement, ~ 2×10^6 cells were suspended in 4 ml of buffer containing (in mM): 136 $NaNO_3$, 3 KNO_3, 2 $Ca(NO_3)_2$, 20 HEPES, 11 Glucose, pH 7.4. Ionophores were dissolved in DMSO to a concentration of 10 mM. After an initial equilibration, chloride efflux was induced by a small volume (<1%) of ionophore. The measurement was concluded with the addition of the sodium dodecyl sulphate (SDS) to break off the membranes and measure the total chloride content in the cells. Experiments were done at 25 ± 1°C.

Supplementary References

1. K. Dairi, S. Tripathy, G. Attardo and J.–F. Lavallée, *Tetrahedron Lett.*, 2006, **47**, 2605–2606; b) D. M. Pinkerton, M. G. Banwell and A. C. Willis, *Org. Lett.*, 2007, **9**, 5127–5130
2. Trofimov, B. A. *et al*, *Chem. Heterocycl. Comp.*, 1985, **21**, 46–49
3. International Tables for Crystallography, Kluwer, Dordrecht, 1992, vol. C.
4. Frisch, M. J.; Trucks, G. W.; Schlegel, H. B.;et al Gaussian 09, Gaussian, Inc., Wallingford CT, 2009
5. (a) Barone, V.; Cossi, M. *J. Phys. Chem. A* **1998**, *102*, 1995. (b) Cossi, M.; Rega, N.; Scalmani, G.; Barone, V.; *J. Comput. Chem*., **2003**, *24*, 669.
6. MacDonald, R. C. *et al.* Small-volume extrusion apparatus for preparation of large, unilamellar vesicles. *Biochim Biophys Acta* **1061,** 297–303 (1991).
7. Galietta, L., Haggie, P. & Verkman, A. Green fluorescent protein-based halide indicators with improved chloride and iodide affinities. FEBS Lett 499, 220–224 (2001).
8. Caci, E. *et al.* Evidence for direct CFTR inhibition by CFTR(inh)-172 based on Arg347 mutagenesis. *Biochem J* **413,** 135–42 (2008).
